# A Toxin Involved in *Salmonella* Persistence Regulates Its Activity by Acetylating Its Cognate Antitoxin, a Modification Reversed by CobB Sirtuin Deacetylase

**DOI:** 10.1128/mBio.00708-17

**Published:** 2017-05-30

**Authors:** Chelsey M. VanDrisse, Anastacia R. Parks, Jorge C. Escalante-Semerena

**Affiliations:** University of Georgia—Athens, Athens, Georgia, USA; University of Michigan—Ann Arbor

**Keywords:** CobB sirtuin deacetylase, lysine acetylation, persistence, protein acetylation, protein synthesis inhibition, type II toxin-antitoxin

## Abstract

Bacterial toxin-antitoxin systems trigger the onset of a persister state by inhibiting essential cellular processes. The TacT toxin of *Salmonella enterica* is known to induce a persister state in macrophages through the acetylation of aminoacyl-tRNAs. Here, we show that the TacT toxin and the TacA antitoxin work as a complex that modulates TacT activity via the acetylation state of TacA. TacT acetylates TacA at residue K44, a modification that is removed by the NAD^+^-dependent CobB sirtuin deacetylase. TacA acetylation increases the activity of TacT, downregulating protein synthesis. TacA acetylation altered binding to its own promoter, although this did not change *tacAT* expression levels. These claims are supported by results from *in vitro* protein synthesis experiments used to monitor TacT activity, *in vivo* growth analyses, electrophoretic mobility shift assays, and quantitative reverse transcription-PCR (RT-qPCR) analysis. TacT is the first example of a Gcn5-related *N-*acetyltransferase that modifies nonprotein and protein substrates.

## IMPORTANCE

During host infection, pathogenic bacteria can modulate their physiology to evade host defenses. Some pathogens use toxin-antitoxin systems to modulate a state of self-toxicity that can decrease their cellular activity, triggering the onset of a persister state. The lower metabolic activity of persister cells allows them to escape host defenses and antibiotic treatments. Hence a better understanding of the mechanisms used by pathogens to ingress and egress the persister state is of relevance to human health.

## INTRODUCTION

Acetylation of proteins and small molecules is a conserved mechanism that regulates cellular processes in cells from all domains of life. Members of the Gcn5-related *N*-acetyltransferase (GNAT) protein superfamily (PF00583) acetylate proteins and non-protein substrates at the expense of acetyl-coenzyme A (Ac-CoA) (1). In some instances, protein acetylation is reversed by class III NAD^+^-dependent deacetylases, also known as sirtuins (2).

The genome of the human pathogen *Salmonella enterica* subsp. *enterica* serovar Typhimurium LT2 (here *S. enterica*) encodes 26 putative GNATs, three of which are annotated as toxin acetyltransferases. These putative toxins are part of type II toxin-antitoxin (TA) systems (3, 4), and the genes encoding these proteins appear to comprise separate operons for each toxin-antitoxin pair.

TA systems have different physiological functions, with some of them contributing to plasmid stabilization as addiction modules (5–9), while others serve as survival management systems under different stress conditions (10, 11). TA systems have been shown to downregulate essential functions that can trigger the onset of a persister state that allows cells to survive unfavorable conditions without the need to acquire mutations (12–17). Importantly, the slowing of essential metabolic processes by TA module expression may allow for tolerance to antimicrobials, which can lead to recalcitrant infections (18, 19).

Type II TA systems are in most cases a part of two-gene operons that encode a protein toxin and protein antitoxin (20). Typically, the antitoxin neutralizes the toxin until a signal induces selective degradation of the antitoxin, often by stress-induced proteases (16, 21–24). Antitoxin degradation creates a stoichiometric imbalance that releases the toxin, thereby increasing its activity and upregulating its transcription (25, 26). With some exceptions, antitoxins possess a DNA-binding domain that recognizes the operator site of its own promoter, resulting in modulation of its own synthesis (11, 27, 28). The *S. enterica* genome encodes three TA systems that include toxins that are homologous to Gcn5-related *N-*acetyltransferases (GNATs). All three of these systems contribute to the onset of a *S. enterica* persister state inside macrophages (29, 30). In addition, Helaine and coworkers showed that the TacT protein of a TA type II system comprised of proteins TacT (STM3651) and TacA (STM3652) acetylates the aminoacyl moiety of several charged tRNAs, arresting translation and triggering a persister state (31). Cheverton et al. suggested that exiting TacT-induced persistence was due to replenishment of tRNA pools through the hydrolysis of the acetylated amino group off of the aminoacyl-tRNA by the peptidyl-tRNA hydrolase Pth (31).

Here we show that, in addition to acetylating aminoacyl-tRNAs, TacT acetylates its cognate TacA antitoxin with a concomitant increase in TacT activity without complex dissociation, a unique attribute not seen with other TA modules. We suggest that the acetylation state of the TacA antitoxin, not its degradation, rapidly modulates TacT activity. We also show that the NAD^+^-dependent CobB sirtuin deacetylase reverses the effect that acetylated TacA has on TacT activity and suggest that reversible TacA acetylation plays a key role in exiting the persister state. In addition, we present evidence that the TacA antitoxin binds to the *tacA-tacT* promoter when in complex with TacT. The involvement of sirtuin-dependent reversible lysine acetylation (sRLA) frames the persister state of *S. enterica* within the carbon and energy statuses of the cell.

## RESULTS

### The absence of TacA antitoxin extends the lag time before the onset of exponential growth in cells with higher levels of *tacT*^*+*^ expression.

**FIG 1:**
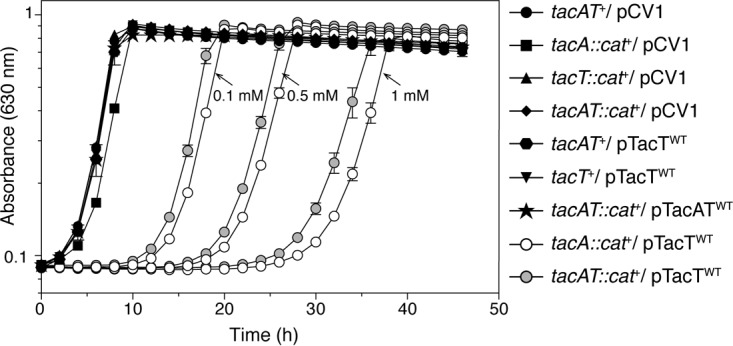
*S. enterica* cells lacking *tacA* and harboring *tacT*^+^ plasmids have growth delays associated with increased expression of *tacT*. Genes coding for TacT (pTacT) or TacAT (pTacAT) were cloned into a complementation vector and introduced into strains with genetic backgrounds identified by the symbols in the graph. Overnight cultures were grown in NB rich medium. Cells were subcultured (1% [vol/vol]) and grown in NCE minimal medium supplemented with glycerol (22 mM) and ampicillin, and transcription of plasmids containing *tacT*^+^ was induced using the levels of arabinose designated by arrows (gray and white circles). All strains with solid black symbols contained 0.15 mM arabinose. Growth curves were obtained in technical triplicates of biological triplicates, and analyses were repeated three times using a microplate reader (Biotek Instruments). Error bars represent standard deviations (SD) from technical triplicates.

Consistent with published data (31), ectopic expression of *tacT*^*+*^ in *S. enterica tacA*::*cat*^*+*^ or *tacAT*::*cat*^*+*^ mutant strains delayed the onset of exponential growth when cells were grown on minimal medium (Fig. 1) due to increased TacT-mediated aminoacyl-tRNA acetylation. This delay was shortened when cells were grown in rich medium, suggesting that nutrient availability played a role in the observed phenotype. Notably, the extended lag phase did not have an effect on the final cell density or growth rate of the cultures (Fig. 1). The phenotype of *tacA*::*cat*^*+*^ strains in which *tacT*^*+*^ was overexpressed (i.e., *tacA*::*cat*^*+*^/pTacT^WT^ or *tacAT*::*cat*^*+*^/pTacT^WT^) was corrected by in *trans* expression of *tacA*^+^ (Fig. 1), indicating that the observed phenotype was dependent on the absence of the TacA antitoxin.

### TacT acetylates residue K44 of TacA.

**FIG 2:**
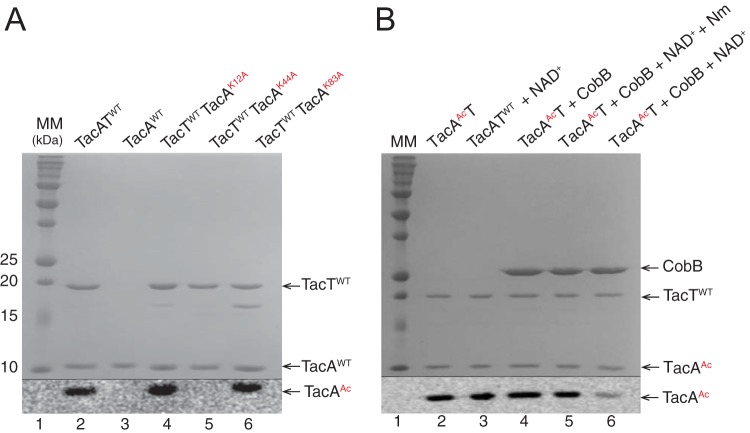
(A) TacA residue K44 is acetylated by TacT. Wild-type TacA-TacT complex (lane 2) or variant (lanes 4, 5, and 6) complexes and TacA (lane 3) were incubated with [1-^14^C]acetyl-CoA. Proteins were separated by SDS-PAGE and stained with Coomassie blue to visualize proteins. Precision Plus protein (Bio-Rad) standard was used as a molecular mass marker (MM). Acetylation was visualized by phosphorimaging. Extra bands in lanes 4 and 5 were analyzed via mass spectrometry and were identified as TacT, which we presumed was cleaved or lacked the hexahistidine tag. (B) TacA^Ac^ is deacetylated by CobB. The reaction mixture used as a positive control contained wild-type TacAT (TacAT^WT^) complex and [1-^14^C]acetyl-CoA (lane 2), positive-control mixture plus NAD^+^ (lane 3), positive-control mixture plus CobB (lane 4), positive-control mixture plus CobB plus NAD^+^ plus Nm (lane 5), or positive-control mixture plus CobB plus NAD^+^ (lane 6). Proteins were separated by SDS-PAGE and stained with Coomassie blue to visualize proteins. Precision Plus protein standard (Bio-Rad) was used as a molecular mass marker (MM [lane 1]). Acetylation was visualized by phosphorimaging. On panels A and B, the thin black line at the bottom of the figures separates the SDS-PAGE gel from the phosphorimage. CobB, sirtuin deacetylase; Nm, nicotinamide; TacT; toxin; TacA, antitoxin.

As shown by others, TacA copurified with TacT, forming a complex. TacA and TacT proteins were synthesized from a vector containing the coding sequences for both proteins. Overexpression of both genes allowed for simultaneous synthesis and folding of each protein, leading to a stable TacAT complex. All toxin-antitoxin complexes (wild-type [WT] and variant proteins) reported herein were copurified as described in Materials and Methods. Our assessment of the purity of the proteins used in this study can be found in Fig. S1 in the supplemental material. Results of gel permeation chromatography experiments revealed the TacAT complex was a dimer of dimers (Fig. S2). When purified TacA and TacT proteins were incubated with [1-^14^C]Ac-CoA, TacA was acetylated (Fig. 2A, lane 2), a modification that required TacT (Fig. 2A, lane 2 versus 3). Liquid chromatography-tandem mass spectrometry (LC-MS/MS) peptide fingerprinting analysis of acetylated TacA (TacA^Ac^) showed that residues K12, K44, and K83 were acetylated to various degrees (Fig. S3). Figure S3 shows peptide masses with different *m*/*z* ratios used search the database using MASCOT software. Analyses of areas under the peaks for peptides corresponding to acetylated K12, K44, and K83 showed a 2.64-fold increase for K12, 18.86-fold increase for K44, and a 3.12-fold increase for K83.

10.1128/mBio.00708-17.1 FIG S1  Percentages of purity of proteins used in these studies. A 15% (wt/vol) SDS-PAGE gel was loaded with ~1 μg of each protein designated above each well. Percentages of purity are as follows: TacAT^WT^, 88%; TacA^K12A^T^WT^, 97%; TacA^K44A^T^WT^, 99%; TacA^K44Q^T^WT^, 99%; TacA^K44R^T^WT^, 99%; TacA^K83A^T^WT^, 85%; TacT, 10%; TacA, 99%; CobB, 99%. Percentage of purity was calculated by diluting proteins (3, 2, 1, 0.5, and 0.25 μg) on a separate gel, and bands were quantified as percentages using ImageQuant TL. Contaminants of the TacT denatured and refolded preparations include ClpB (ATP-dependent chaperone), HtpG (molecular chaperone), MdaA (NADPH nitroreductase), elongation factor Tu, peptidyl-prolyl *cis*-*trans* isomerase, ferric uptake regulation protein, transposase, and YdaA (uncharacterized protein). MM, molecular mass marker. Download FIG S1, TIF file, 11.8 MB. Copyright © 2017 VanDrisse et al. 2017 VanDrisse et al. This content is distributed under the terms of the Creative Commons Attribution 4.0 International license.

10.1128/mBio.00708-17.2 FIG S2  Size exclusion chromatography of purified complexes. The molecular masses of TacAT^WT^, TacA^K44R^T^WT^, and TacA^K44Q^T^WT^ were determined by size exclusion chromatography using a Superose 12 10/300 GL column as detailed in Materials and Methods. The molecular mass standards (gray circles) were thyroglobulin (bovine [670,000 Da]), gamma globulin (bovine [158,000 Da]), ovalbumin (chicken [44,000 Da]), myoglobin (horse [17,000 Da]), and vitamin B_12_ (1,350 Da). Download FIG S2, TIF file, 2 MB. Copyright © 2017 VanDrisse et al. 2017 VanDrisse et al. This content is distributed under the terms of the Creative Commons Attribution 4.0 International license.

10.1128/mBio.00708-17.3 FIG S3  Mass spectrometry analysis of acetylated TacA. The sequence at the top represents annotated TacA protein sequences. Residues in gray represent amino acids not detected by LC-MS/MS, and therefore the start methionine was repositioned to begin with black sequence. Residues identified as being acetylated in TacAT^WT^ samples containing 1 mM acetyl-CoA are highlighted in red. b ions are the series of fragments that extend from the amino terminus, and y ions are the series of ions that extend from the C terminus. MASCOT software was the online search engine used to identify peptides on the basis of their masses. Ion series a, b, and y are the expected ion types. The subscript of an a, b, or y ion represents the number of residues in the peptide, and the superscript represents the peptide charge. Download FIG S3, TIF file, 15.5 MB. Copyright © 2017 VanDrisse et al. 2017 VanDrisse et al. This content is distributed under the terms of the Creative Commons Attribution 4.0 International license.

### Correction of the translation initiation codon of TacA.

In the *Salmonella* genome, *tacAT* comprise an operon with *tacA* being promoter proximal, with the last 13 bp of *tacA* overlapping the *tacT* coding sequence. The 5′ region of *tacA* contains the annotated start methionine and an additional methionine at position 8. This information was relevant, because the above-mentioned peptide fingerprinting analysis did not detect the first 7 amino acids (M1 to L7 [gray residues in Fig. S3]) of the annotated primary sequence of TacA; therefore, we considered the possibility that the true start methionine of TacA was residue M8. This misannotation became relevant during the purification of TacAT complexes, because the nucleotides encoding the first 7 amino acids and the *S*-tag (streptavidin tag) fused to TacA were not translated, resulting in coelution of tagless native TacA with H_6_-TacT. The availability of tagless TacA became useful for the performance of DNA binding experiments, because the DNA-binding domain of TacA was on its N terminus. For the experiments mentioned above and from here on, the translation start codon of TacA was reassigned to the ATG encoding M8. That is, residue M8 became M1, and the numbering for the rest of the residues was modified accordingly. Consequently, residues K19, K51, and K90 became K12, K44, and K83, respectively. The adjusted numbering was used throughout these studies.

### Residue K44 is a key acetylation site in TacA, a modification reversed by the NAD^+^-dependent CobB sirtuin deacetylase.

To validate the putative acetylation sites, TacA variants with substitutions at positions K12, K44, and K83 were isolated. As shown in Fig. 2A, transfer of the acetyl moiety of [^14^C]Ac-CoA was not observed when TacT was in complex with TacA^K44A^, indicating that either K44 was the only acetylation site in TacA that was modified by TacT or K44 acetylation triggered K12 and K83 acetylation by TacT. The latter scenario was not pursued. Instead we focused on the question of whether or not K44 acetylation was reversible. In *Salmonella*, the only known protein lysine deacetylase is the NAD^+^-dependent CobB sirtuin. This result raised the question of whether CobB could deacetylate TacA^Ac^. To test this idea TacA^[14C]Ac^ was incubated with CobB, NAD^+^, CobB plus NAD^+^, or CobB plus NAD^+^ plus nicotinamide (Nm). Under the conditions tested, CobB and NAD^+^ deacetylated ~70% of TacA^Ac^ (Fig. 2B, lane 6 versus lanes 2, 3, 4, and 5). As expected, CobB activity was inhibited by Nm (Fig. 2B, lane 5 versus 6), a result consistent with CobB-dependent deacetylation.

### TacA acetylation enhances TacT activity *in vitro*.

TacA variants were constructed to determine whether or not acetylation of TacA residue K44 had an effect on TacT-dependent arrest of mRNA translation. TacA variants with substitutions at position K44 that mimicked acetylation (i.e., K44Q) or deacetylation (i.e., K44R) (2) were isolated in complex with wild-type TacT (TacT^WT^). No effect on the formation or stability of complexes between TacT^WT^ and TacA variants was detected using gel filtration chromatography (Fig. S2). TacT was purified from homogeneous TacAT complex by denaturation and refolding, as described in Materials and Methods.

**FIG 3:**
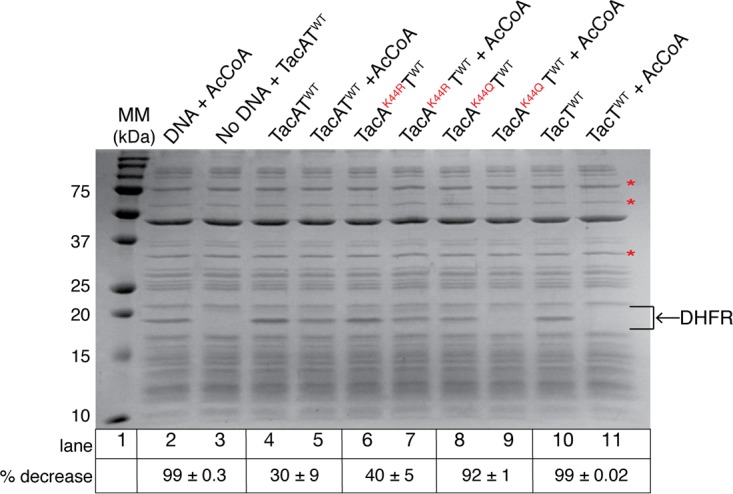
*In vitro* DHFR protein synthesis used to quantify aminoacyl-tRNA acetylation by TacT shows TacA acetylation variants increase TacT-mediated aminoacyl-tRNA acetylation. The SDS-PAGE gel shows *in vitro* synthesis of dihydrofolate reductase (DHFR [indicated by arrow]) in reaction mixtures containing the additions indicated above each lane. Precision Plus protein standard (Bio-Rad) was used as a molecular mass marker (MM [lane 1]). Lane 2 represents a positive control for DHFR production, where the reaction mixture contained DHFR DNA (100 ng) and acetyl-CoA to ensure acetyl-CoA stocks did not interfere with reaction components. The reaction mixture in lane 3 lacked DNA and contained TacAT^WT^ complex to control for any added bands that might interfere with interpretations. Lanes 4 to 9, reaction mixtures that contained TacAT^WT^ or variant complexes (indicated by labels above each lane) with or without acetyl-CoA (with DNA added to lanes 4 to 11). Lanes 10 and 11, positive control for aminoacyl-tRNA acetylation. These reaction mixtures contained TacT with or without acetyl-CoA. All reaction mixtures were incubated for 2 h at 37°C. Details of the procedure can be found in Materials and Methods. Asterisks represent bands used to normalize the intensity of the DHFR protein within each lane (ImageQuant v5.2 software). Comparison of DHFR band intensity to bands with asterisks was averaged, and the standard deviation of these three numbers was calculated. The percentage of decrease with SD for each sample is indicated below the corresponding lanes.

*In vitro* protein synthesis experiments were performed with TacAT^WT^, TacA^K44Q^T^WT^, and TacA^K44R^T^WT^ complexes or TacT^WT^ alone. Proteins were incubated with or without Ac-CoA in a cell-free protein synthesis system that contained all machinery necessary for transcription and translation from a synthesized DNA product. DNA that is added to the reaction mixtures codes for dihydrofolate reductase (DHFR), and its synthesis is used as a reporter of mRNA translation. DHFR synthesis was monitored using SDS-PAGE (32) (~18 kDa [arrow in Fig. 3]). TacT^WT^ toxin was expected to acetylate aminoacyl-tRNAs, resulting in DHFR protein synthesis inhibition (31). To quantify differences in DHFR synthesis in reaction mixtures containing or lacking Ac-CoA, the intensities of three bands in each lane (asterisks in Fig. 3) were expressed as percentages of the band intensity of DHFR. For example, the level of DHFR synthesis was compared in reaction mixtures containing TacAT^WT^ with or without Ac-CoA (Fig. 3, lanes 4 and 5). Control experiments for these studies included conditions for maximal DHFR synthesis (Fig. 3, lane 2), with the baseline for the production of DHFR being established by a reaction mixture devoid of DNA. As expected, no DHFR was synthesized (<1%) in the absence of added DHFR-encoding DNA (Fig. 3, lane 2 versus 3). Two reaction mixtures lacking TacA^WT^, with or without Ac-CoA added, showed TacT^WT^- and Ac-CoA-dependent arrest of DHFR synthesis (Fig. 3, lanes 10 versus 11), indicating that the TacT^WT^ protein was active.

DHFR synthesis was reduced by ~30% in reaction mixtures containing TacAT^WT^ in the presence of Ac-CoA (Fig. 3, lane 4 versus 5). This result was unexpected because, to our knowledge, this is the first report of a toxin suggested to be active in complex with its cognate antitoxin. Reaction mixtures containing TacA^K44R^T^WT^ (deacetylation mimic variant) and acetyl-CoA showed a 40% reduction in DHFR synthesis (Fig. 3, lane 6 versus 7). Although it appears there are differences in aminoacyl-tRNA acetylation by the TacAT^WT^ and TacA^K44R^T^WT^ proteins, these differences are not statistically significant (see percentages as standard deviations in Fig. 3). In sharp contrast, DHFR synthesis was reduced by 92% in reaction mixtures containing the acetylation mimic variant TacA^K44Q^T^WT^ complex, and Ac-CoA (Fig. 3, lane 8 versus 9), suggesting that the TacA^K44Q^ variant enhanced the acetylation of aminoacyl-tRNA by TacT^WT^ (Fig. 3, lane 8 versus 9). These results strongly suggested that TacA^WT^ acetylation by TacT^WT^ upregulated TacT^WT^-dependent acetylation of aminoacyl-tRNAs. A summary of the above results is presented as percentages underneath the SDS-PAGE gel (Fig. 3).

### TacA acetylation enhances TacT activity *in vivo*.

**FIG 4:**
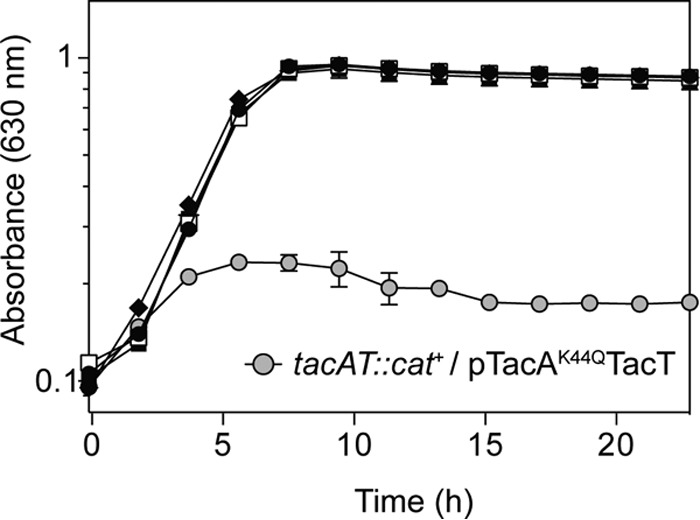
TacA variant mimicking acetylation in complex with TacT in *S. enterica tacAT*::*cat*^+^ strains causes growth defect. Genes coding for TacT^WT^ (pTacT), TacAT^WT^ (pTacAT^WT^), TacA^K44R^T^WT^ (pTacA^K44R^TacT^WT^), and TacA^K544Q^T^WT^ (pTacA^K44Q^TacT^WT^) were cloned into a complementation vector (pCV1) and introduced into the strain backgrounds indicated by the following symbols: *tacAT*::*cat*^*+*^/pCV1 (empty cloning vector control), solid circles; *tacAT*::*cat*^+^/pTacT^WT^, open squares; *tacAT*::*cat*^+^/pTacAT^WT^, solid squares; *tacAT*::*cat*^+^/pTacA^K44R^T^WT^, diamonds; *tacAT*::*cat*^+^/pTacA^K44Q^T^WT^, gray circles. Overnight cultures were grown in NB rich medium. Cells were subcultured (1% [vol/vol]) and grown in NCE minimal medium supplemented with glycerol (22 mM) and ampicillin. Expression of plasmid-borne genes was induced with 25 μM l-(+)-arabinose. Growth curves were obtained in technical triplicates of biological triplicates, and analyses were repeated three times using a microplate reader (Biotek Instruments). Error bars represent standard deviations from technical triplicates.

To validate *in vitro* results *in vivo*, *tacA* alleles encoding TacA variants with single-amino-acid substitutions at position K44 were introduced by site-directed mutagenesis into a plasmid carrying *tacAT*^*+*^ (pTacAT-1). One TacA variant within the operon had a K44Q substitution (TacA^K44Q^) for the purpose of mimicking acetylation (pTacAT-10), another variant had a K44R substitution (TacA^K44R^) to mimic deacetylation (pTacAT-9). The resulting plasmids, which also carried the *tacT*^*+*^ allele, were individually introduced into *tacAT*::*cat*^*+*^ strains. As shown in Fig. 4, *tacAT*::*cat*^*+*^ strains that synthesized TacA^K44Q^T^WT^ complex had a striking growth phenotype compared to strains making TacA^K44R^T^WT^ (Fig. 4, gray circles and black diamonds). This result was consistent with an increase in aminoacyl-tRNA acetylation by TacT^WT^ that slowed down mRNA translation, causing the observed phenotype. We note that the growth arrest of strains making TacA^K44Q^T^WT^ was more severe than the growth delay of strains expressing *tacT*^*+*^ alone and is most likely due to lower induction of plasmids coding for TacA^K44Q^T^WT^ causing this phenotype (i.e., overexpression of *tacT* alone at 25 μM arabinose is not a high enough induction to cause a growth delay). This is most likely due to stability of the toxin when it is in or not in complex with its antitoxin, and these results are further discussed below.

### Acetylation of residue K44 of TacA alters the DNA binding of the protein.

Electrophoretic mobility shift assays (EMSAs) were performed to determine whether modifications of K44 would affect the DNA-binding activity of TacA. A 157-bp 6-carboxyfluorescein (6-FAM) 5′-labeled probe (positions −163 to −6 [probe 1]) upstream of the corrected ATG transcription initiation codon for *tacAT* was chosen for probe design. TacAT^WT^ complex bound to this probe, and this binding was specific because TacAT did not bind to the promoter for *argS*, a negative control (see Fig. S4 in the supplemental material). To narrow down the minimal sequence necessary for TacAT binding, two probes within the 157-bp region upstream of the putative ATG codon for *tacAT* were designed. One probe was 71 bp long (positions −163 to −92 [probe 2]), and the other was 75 bp long (positions −81 to −6 [probe 3]) (probes 2 and 3 in Fig. S5). There was no shift seen with probe 2, but the electrophoretic mobility of the 75-bp probe (probe 3) changed in the presence of the TacAT^WT^ complex (Fig. S5).

10.1128/mBio.00708-17.4 FIG S4  TacAT^WT^ complexes bind specifically to the *tacAT* promoter. The specificity of binding of TacAT^WT^ complexes to the *tacAT* promoter was analyzed by electrophoretic mobility shift assays using 6-FAM 5′-labeled probes. (Left half) Probe 1 (157 bp, 0.48 pmol, positions −163 to −6) and competitor DNA (*argS* promoter DNA, 0.38 pmol, 196 bp) were incubated together with increasing concentrations of TacAT^WT^ complex (0.24, 0.48, 1.45, 2.4, and 4.8 pmol protein to DNA). (Right half) *argS* promoter DNA (0.38 pmol) incubated with TacAT^WT^ at increasing molar fold excess (0.54, 1.08, 3.25, 5.41, and 10.8 pmol). Download FIG S4, TIF file, 2.4 MB. Copyright © 2017 VanDrisse et al. 2017 VanDrisse et al. This content is distributed under the terms of the Creative Commons Attribution 4.0 International license.

10.1128/mBio.00708-17.5 FIG S5  TacA-TacT complexes bind to a specific region within the *tacAT* promoter. An attempt to narrow down the binding region of the TacAT^WT^ complex within the *tacAT* promoter was conducted using electrophoretic mobility shift assays with 6-FAM 5′-labeled probes. (Top panel, left) A fragment of DNA consisting of the upstream 71 bp of probe 1 called probe 2 (1.06 pmol, 71 bp) was incubated with increasing concentrations of TacAT^WT^ complex (protein added to probe 2 at 0.53, 1.06, 3.2, 5.3, and 10.6 pmol). (Top panel, right) TacAT^WT^ complex (0.5, 1.01, 3.03, 5.05, and 10.1 pmol) was incubated with the downstream 75 bp of probe 1 called probe 3 (1.01 pmol). (Bottom panel) To determine if two binding sites exist within probe 3, the sequence of probe 3 was then divided in half to form probe 4 (upstream 35-bp region, 2.16 pmol) and probe 5 (downstream 40-bp region, 1.89 pmol). TacAT^WT^ complex was incubated with either probe 4 or probe 5 to show binding to two different sites at molar fold excesses of 1.08, 2.16, 6.49, 10.8, and 21.6 pmol protein to probe 4 and 0.95, 1.89, 5.68, 9.5, and 18.9 pmol protein to probe 5. The DNA sequence represents −163 bp to ATG of *tacA*. The ATG start codon matches the correct reading frame as mentioned in the Results section. Download FIG S5, TIF file, 17.7 MB. Copyright © 2017 VanDrisse et al. 2017 VanDrisse et al. This content is distributed under the terms of the Creative Commons Attribution 4.0 International license.

TacA^WT^ or TacT^WT^ alone did not bind to probe 3 (see Fig. S6A in the supplemental material). This result suggested that TacA needed TacT to bind to DNA. An electrophoretic mobility shift assay (EMSA) was conducted in which TacA and TacT proteins were present to test whether or not they could reform a complex capable of binding to probe 1. No binding was detected at 5-fold excess TacA and 50-fold excess TacT, nor was DNA binding observed when TacA protein was incubated with probe 1 at 50-fold excess (Fig. S6B). We concluded that refolded TacT was active based on the results presented in Fig. 3. However, we cannot rule out the possibility that TacA did not refold correctly, thus preventing DNA binding. It is also possible that the complex could not reform after TacA and TacT were separated.

10.1128/mBio.00708-17.6 FIG S6  TacA and TacT alone do not bind to the *tacAT* promoter. (A) To test whether or not TacA^WT^ could bind to its own promoter in the absence of TacT^WT^, probe 3 and TacA^WT^ protein were incubated together at 1.01, 3.03, 5.05, 10.1, and 15.15 molar fold excess protein to probe (1.01 pmol). As a control to ensure that TacT^WT^ alone had no DNA binding activity, TacT^WT^ protein was incubated with probe 3 at the same molar fold excess stated above. (B) To test whether TacA and TacT^WT^ protein could reform a complex and bind to DNA, probe 1 (0.24 pmol) was incubated with either 5 molar-fold excess TacAT^WT^ complex, 5-fold excess TacA^WT^, 50-fold excess TacT^WT^, or mixed 5-fold excess TacA^WT^ and 50-fold excess TacT^WT^. Fifty-fold excess TacT^WT^ was used because TacT^WT^ was 10% pure (Fig. S1). Download FIG S6, TIF file, 8.2 MB. Copyright © 2017 VanDrisse et al. 2017 VanDrisse et al. This content is distributed under the terms of the Creative Commons Attribution 4.0 International license.

**FIG 5:**
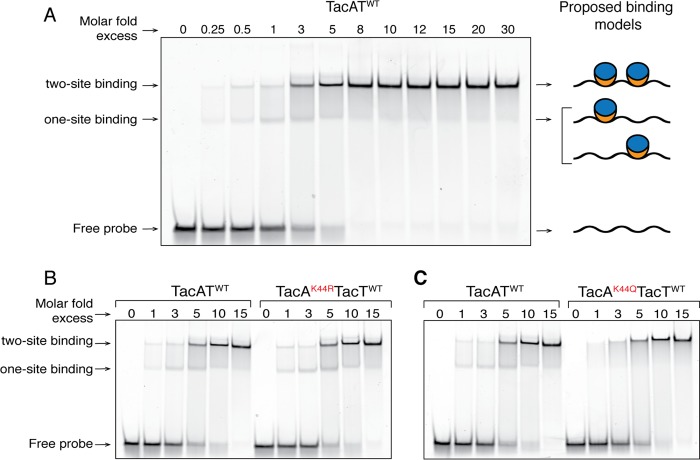
Binding of TacAT^WT^ to the *tacAT* promoter differs from binding of TacA^K44Q^T^WT^. Binding of increasing concentrations of TacAT complexes to the *tacAT* promoter was assessed by electrophoretic mobility shift assays using 6-FAM 5′-labeled probes. (A) Probe 3 (75 bp, 1.01 pmol, positions −81 to −6) was incubated with increasing concentrations of TacAT^WT^ protein (0.25, 0.5, 1.01, 3.03, 5.05, 8.08, 10.1, 12.12, 15.15, 20.2, and 30.3 pmol). The diagram on the right side of the figure depicts a model that could explain the occurrence of the two shifted bands seen (TacT, blue ovals; TacA, orange crescents). (B) Probe 3 (1.06 pmol) incubated with increasing concentrations of either TacAT^WT^ or TacA^K44R^T^WT^ (molar excesses of both complexes at 0.53, 1.06, 3.2, 5.3, and 10.6 pmol). (C) Probe 3 (1.06 pmol) incubated with TacAT^WT^ or TacA^K44Q^T^WT^ at the same molar fold excesses as panel B.

We observed two different probe shifts (Fig. 5A, labeled with one- and two-site binding), which were not due to a mixed population of complex (i.e., acetylated versus non-acetylated) since a TacA^K44R^T^WT^ complex (i.e., not the acetylatable variant, but a positively charged substitution of K44) produced the same two shifts (Fig. 5B). To ascertain whether the observed two bands reflected the presence of two binding sites within probe 3, the probe was split into two pieces: probes 4 and 5 (sequence representation in Fig. S5). Probe 4 (−81 to −46) or probe 5 (−46 to −6) was mixed with TacAT^WT^ complex, and interactions were assessed by EMSAs (Fig. S5). The two bands observed when probes 1 and 3 were used (Fig. 5; Fig. S5) were not observed when probes 4 and 5 were used (Fig. S5), indicating that only one binding site was present in each probe. Work on the MqsRA TA system in *Escherichia coli* showed that two band shifts occur when two binding sites are present (46). Such a scenario is consistent with our observations (Fig. 5A) in that the lower band assigned to TA complex bound to either site and the upper band reflecting interactions of complexes bound to two sites (Fig. 5A, cartoon representation).

In contrast to the TacA^K44R^T^WT^ complex, the TacA^K44Q^T^WT^ complex (i.e., mimicking acetylated K44) did not display the second band, in support of the idea that TacA^K44Q^T^WT^ binds to both sites (Fig. 5). These data suggested that residue K44 did not directly affect the DNA-binding activity of TacA, but that acetylation altered TacA recognition of or binding to the *tacAT* promoter. It was further validated that K44 was not directly involved in DNA binding because the TacA^K12A^T^WT^ complex had DNA binding activity that was abrogated (Fig. S7), suggesting the N terminus included the DNA-binding domain of TacA. In contrast to this finding, the binding levels of TacA^K83A^T^WT^ and TacAT^WT^ complexes to probe 3 were very similar, suggesting that residue K83 and therefore the C terminus was not involved in TacA binding to DNA. A predicted structural representation of these residues can be seen in Fig. S8.

10.1128/mBio.00708-17.7 FIG S7  Residue K12 of TacA is needed to bind DNA. The ability of lysine variants TacA^K12A^T^WT^ and TacA^K83A^T^WT^ to bind to DNA was analyzed by EMSA. Probe 3 (1.01 pmol) was incubated with increasing molar excess of either TacA^K12A^T^WT^ or TacA^K83A^T^WT^ (0.5, 1.01, 3.03, 5.05, and 10.1 pmol protein). Download FIG S7, TIF file, 6.3 MB. Copyright © 2017 VanDrisse et al. 2017 VanDrisse et al. This content is distributed under the terms of the Creative Commons Attribution 4.0 International license.

10.1128/mBio.00708-17.8 FIG S8  Schematic of SOE-qPCR fragment assembly as described in Materials and Methods. The upstream region in red is comprised of 792 bp upstream of *tacAT*. The downstream region is comprised of ~1 kb of *tacAT*. Fragments were fused together using SOE-qPCR with the primers listed in Table S2. DNA sizes are to scale. Download FIG S8, TIF file, 10.6 MB. Copyright © 2017 VanDrisse et al. 2017 VanDrisse et al. This content is distributed under the terms of the Creative Commons Attribution 4.0 International license.

**FIG 6:**
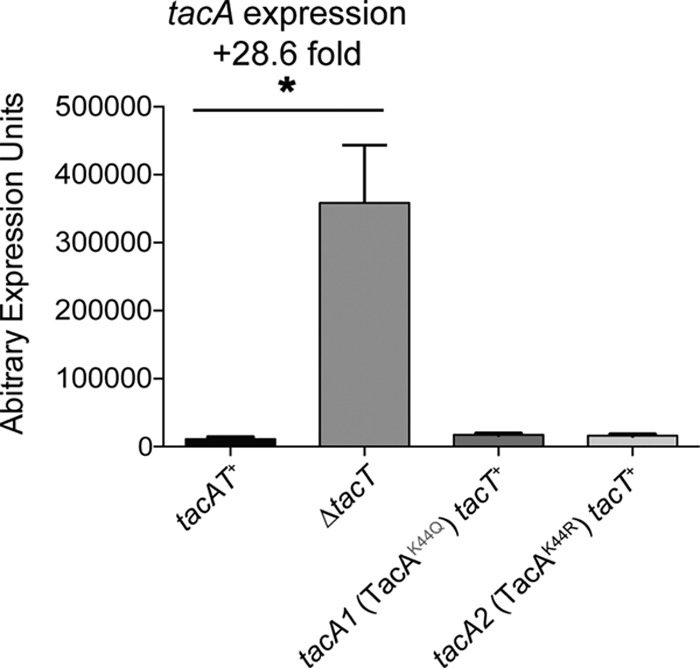
Deletion of *tacT* relieves repression of *tacA*. RT-qPCR was used to evaluate the expression of the *tacA* gene in the backgrounds of *tacAT*^*+*^, Δ*tacT*, *tacA1 tacT*^*+*^ (TacA^K44Q^), and *tacA2 tacT*^*+*^ (TacA^K44R^) under the condition of NCE minimal medium supplemented with glycerol (22 mM). The gene expression of *tacA* in the toxin deletion strain Δ*tacT* was 28.6-fold higher than that in the *tacAT*^*+*^ strain. Interestingly, the two lysine variant strains yielded no significant difference in expression of *tacA* compared to the *tacAT*^*+*^ strain. Error bars represent SEM. An asterisk indicates that Welch’s *t* test gave a *P* value of 0.01. The experiment was conducted in technical triplicates of biological triplicates and was repeated three times.

To determine whether the altered binding to DNA by TacA variants in complex with TacT^WT^ had an effect on repression of *tacAT* transcription, chromosomal mutations coding for K44R or K44Q TacA variants were constructed as described in Materials and Methods. Cells coding for TacA^WT^T^WT^, TacA^K44R^T^WT^, or TacA^K44Q^T^WT^ or cells lacking TacT were grown to mid-log phase on minimal medium supplemented with 22 mM glycerol, and total RNA was extracted as described previously (33). We performed RT-qPCR with total RNA to measure the differences in level of mRNA transcript of the *tacA* gene in the mutants compared to the *tacAT*^+^ strain. We observed a large increase in *tacA* transcript (28.6-fold) in the strain in which the *tacT* gene was deleted and only *tacA* remained (Fig. 6). This suggested that the native TacAT complex repressed the operon under this condition and that the presence of TacT was needed for TacA-mediated repression. When measuring *tacA* transcript in the chromosomal variants, we did not detect significant transcript differences between the TacA^K44R^T^WT^ or TacA^K44Q^T^WT^ strain and the parent strain. These results suggested either that the altered DNA binding activity of TacA^K44Q^T variants seen *in vitro* might be an artifact of the assay or that an additional factor for *tacAT* expression might be needed and may only be induced under different conditions.

## DISCUSSION

Here we report insights into the functionality of one of the three type II toxin-antitoxin systems encoded by the genome of *Salmonella enterica*, a human pathogen. The *S. enterica* TacAT system (encoded by *tacA* and *tacT* [formerly STM3651 and STM3652, respectively]) is the focus of the studies reported herein. The TacAT system is the first example of a toxin-antitoxin system whose function is not regulated by dynamic association/dissociation of its components and the first example of a system in which the toxin is a GNAT that recognizes a protein and non-protein substrate. In the case of TacAT, TacT acetylates the α-amino group of aminoacyl-tRNAs (29) and also acetylates a lysine residue of TacA (this work). Furthermore, we have shown that sirtuin-dependent reversible lysine acetylation (sRLA) deacetylates TacA^Ac^ and may play a role in controlling the activities of TacA and TacT *in vivo*.

### A new role for sRLA in *Salmonella* pathogenesis.

We hypothesize that the *S. enterica* TacAT system is different from other type II toxin-antitoxin systems in that the activity of TacT is not upregulated as a result of the cleavage or cellular degradation of the cognate antitoxin (i.e., TacA) (22–24). Our data support the conclusion that TacAT activities are modulated posttranslationally through sRLA (Fig. 3 and 4). The involvement of sRLA in the modulation of the entrance or egress into or out of a persister state suggests a metabolic link between the latter and the carbon (Ac-CoA) and energy (NAD^+^) statuses of the cell (see below).

Although it is clear that acetylation of TacA alters binding of TacA to the two sites present inside the *tacAT* promoter *in vitro* (Fig. 5), more work is needed to understand why and how TacA^Ac^ recognition of two sites is favored over recognition of either one of the two sites and whether or not there are additional factors involved in *tacAT* expression.

### Other unique features of the *S. enterica* TacAT system.

As stated above, a unique feature of TacT is that it can acetylate non-protein and protein substrates. Also unique is the fact that TacT activity is enhanced as a result of the acetylation of TacA by TacT while the proteins are in complex. At present, there is no indication that TacA and TacT ever dissociate from each other. In fact, the aminoacyl-tRNA-acetylating activity of TacT is enhanced when TacT is in a complex with TacA^K44Q^ (an acetylation mimic) (Fig. 3). In contrast, TacT activity decreases in complexes containing TacA or TacA^K44R^ (a deacetylated mimic).

### *In vivo* evidence supports the idea that TacA acetylation enhances TacT activity.

Results from *in vivo* experiments are consistent with *in vitro* results. For example, the phenotype associated with the synthesis of TacA^K44Q^T^WT^ occurred even at low levels of induction (25 μM arabinose), whereas the phenotype generated by a high level of expression of TacT alone was erratic, and when it was observed, it occurred only at high levels of induction (150 to 1,000 μM arabinose). These differences could be due to an increased turnover rate or to poor solubility of TacT in the absence of TacA. Therefore, when TacT is in complex with TacA^K44Q^, TacT is not only stabilized, but its activity is increased, leading to growth arrest (Fig. 4). This result suggests that TacA^Ac^T^WT^ blocks protein synthesis very efficiently. We posit that interaction of *Salmonella* with the macrophage leads to increased Ac-CoA levels as a result of the inhibition of Ac-CoA-consuming processes. Such an increase in Ac-CoA could trigger TacA acetylation by TacT, increased stability of the complex, and increased TacT activity, ultimately resulting in the arrest of protein synthesis. These ideas await further experimentation.

### Additional perspective.

One central, unanswered question is how *S. enterica* monitors and modulates the levels of TacA^WT^T^WT^ and TacA^Ac^T^WT^ complexes. Our data suggest that the NAD^+^-dependent CobB protein deacetylase prevents the accumulation of TacA^Ac^T^WT^ complex that would drive the cell into a prolonged persister state. Given that CobB can deactylate TacA^Ac^ and that NAD^+^ is needed for CobB activity, a plausible answer to the above question may lie on the intracellular NAD^+^ level. A decrease in the Ac-CoA level could be due to its consumption by anabolic processes, which would require a robust energy charge and would correlate with increased NAD^+^ levels in the cell. NAD^+^ would then be used by CobB to reduce TacT activity as a result of TacA^Ac^ deacetylation.

### Importance.

The TacAT toxin-antitoxin system of *S. enterica* appears to have evolved to use sirtuin-dependent reversible lysine acetylation (sRLA) as a mechanism to rapidly enter and leave a persister state. There are several methods of other type II TA systems that use transcriptional regulation to either replenish pools of antitoxin to neutralize toxin activity, as with HicAB (34), or more commonly use the toxin as a corepressor until a stoichiometric imbalance occurs to derepress the operon (known as conditional cooperativity) (35, 36). With the TacAT system, the use of sRLA could quickly and efficiently modulate toxin and antitoxin activities without the need for threshold derepression of transcription. By maintaining a stable TacAT complex, the cell may enter or egress the persister state as a function of Ac-CoA, NAD^+^, and probably other as yet unknown signals. Additional evidence for the involvement of sirtuins in the control of TacAT activities inside the macrophage is needed to advance our understanding of the persister state and how *S. enterica* gets into and out of it. Regardless, the new knowledge reported here suggests a new use of sirtuin activators to help reduce the probability of *S. enterica* maintaining a persister state inside a host.

## MATERIALS AND METHODS

### Bacterial strains.

All strains constructed were derivatives of *Salmonella enterica* subsp. *enterica* serovar Typhimurium LT2 (here *S. enterica*) using the Wanner in-frame gene deletion method (37). Bacteria were grown shaking at 37°C, and the media used for growth are described under “Culture media and chemicals.”

### Culture media and chemicals.

All of the bacterial strains and plasmids used are listed in Table S1 in the supplemental material. Strains were grown in lysogenic broth (LB [Difco]), nutrient broth (NB [Difco]), or no-carbon essential (NCE) minimal medium (38). Growth studies in which glycerol was the sole carbon and energy source were performed in NCE medium supplemented with MgSO_4_ (1 mM), Wolfe’s trace minerals (1×) (39), and glycerol (22 mM). When used, antibiotics were added at the following concentrations: ampicillin, 100 μg/ml; chloramphenicol, 20 μg/ml; and kanamycin, 50 μg/ml. Cultures used as an inoculum were grown overnight at 37°C in NB, and small samples (1% [vol/vol]) were used to inoculate 198 μl of fresh medium placed in each well of a 96-well microtiter plate. l-(+)-Arabinose was used as an inducer wherever indicated. Microtiter plates were incubated at 37°C inside the temperature-controlled chamber of a PowerWave microtiter plate reader (Bio-Tek Instruments), and plates were continuously shaken using the medium setting of the instrument. Cell density was monitored at 630 nm, and data were analyzed using Prism 6 software package (GraphPad).

10.1128/mBio.00708-17.9 TABLE S1  Strains and plasmids used in this work. Download TABLE S1, DOCX file, 0.1 MB. Copyright © 2017 VanDrisse et al. 2017 VanDrisse et al. This content is distributed under the terms of the Creative Commons Attribution 4.0 International license.

### Strain construction.

All strains constructed were *ara-9* derivatives of *S. enterica* using the Wanner in-frame gene deletion method (37). The primers used in this study were synthesized by Integrated DNA Technologies, Inc. (IDT, Coralville, IA), and are listed in Table S2 in the supplemental material. *tacA*::*cat*^+^ and Δ*tacA* strains were engineered as follows. Using Pfu Ultra II Fusion DNA polymerase (Stratagene), flanking regions of plasmid pKD3 (37) were amplified with primers designed with 36 to 39 bp of overlapping region at the beginning of either the *tacA* or *tacT* gene and with 50 bp of overlapping region at the end of the *tacA* or *tacT* gene. PCR fragments were analyzed by agarose gel electrophoresis on 1% (wt/vol) agarose gels poststained with 0.5 μg/ml ethidium bromide for 15 min. PCR fragments were PCR cleaned up using the Wizard SV gel and PCR cleanup system (Promega), and 5 to 10 μl of product was electroporated into *S. enterica* strain JE10813 Δ*ara-9* harboring plasmid pKD46 (37). After electroporation, cells were grown up to an optical density at 630 nm (OD_630_) of ~0.6 at 30°C, followed by three washes with glycerol (10% [vol/vol]). Electroporation was performed in 0.2-cm electroporation cuvettes (MidSci) in a Bio-Rad MicroPulser electroporator on the Ec2 setting. Cells were then recovered in 0.5 ml of LB for 1 h at 37°C, plated on LB plus agar plus antibiotic, and incubated overnight at 37°C. Drug-resistant transformants were streaked repeatedly on antibiotic plates at 42°C to cure the strains of plasmid pKD46. Strains were then reconstructed by P22-mediated transduction of the drug marker into strain JE10079. Strains containing chloramphenicol insertions were transformed with plasmid pCP20 to resolve out the chloramphenicol insertion and make a scarred deletion.

10.1128/mBio.00708-17.10 TABLE S2  Primers and DNA probes used in this study. Download TABLE S2, DOCX file, 0.2 MB. Copyright © 2017 VanDrisse et al. 2017 VanDrisse et al. This content is distributed under the terms of the Creative Commons Attribution 4.0 International license.

### Plasmid construction for complementation and overexpression.

All plasmids used in this work are listed in Table S1. Primers used in this study were synthesized by Integrated DNA Technologies, Inc. (IDT [Coralville, IA]), and are listed in Table S2. We used the high-efficiency cloning method described elsewhere (40) to clone the *tacA*, *tacT*, *tacAT*, and *cobB* genes into pCV1 and pCV3 vectors. Plasmid pCV1 is a modified plasmid of pBAD24 (41) with BspQI sites added and confers ampicillin resistance, and expression of genes cloned into it can be induced with l-(+)-arabinose. Plasmid pCV3 is a modified plasmid of pBAD33 (41) with added BspQI sites and confers chloramphenicol resistance and arabinose induction. Genes *tacA*, *tacT*, *tacAT*, and *cobB* were amplified from the *S. enterica* chromosome using Pfu Ultra II fusion DNA polymerase (Stratagene). PCR fragments were analyzed by agarose gel electrophoresis on 1% (wt/vol) agarose gels stained with ethidium bromide. PCR fragments were PCR cleaned up using the Wizard SV gel and PCR cleanup system (Promega) and digested with the restriction enzyme BspQI (NEB) at 50°C for 1 h; products were ligated with T4 DNA ligase (Fisher).

The overexpression vector pACYCDuet (EMD Millipore Biosciences) possesses two multiple-cloning sites (MCSs) for which two separate genes may be cloned into and overexpressed simultaneously from the same vector. This vector was used for the overexpression of TacT and TacA from the same plasmid. pACYCDuet was first digested with FastDigest (Thermo, Fisher Scientific) BamHI and EcoRI for 1 h at 37°C. *tacA* and *tacT* were amplified from the *S. enterica* chromosome using Pfu Ultra II fusion DNA polymerase (Stratagene). PCR fragments were analyzed by agarose gel electrophoresis on 1% (wt/vol) agarose gels stained with ethidium bromide. PCR fragments were PCR cleaned up using the Wizard SV gel and PCR cleanup system (Promega). The *tacT* PCR product was digested with FastDigest (Thermo, Fisher Scientific) BamHI and EcoRI for 1 h at 37°C. The digested PCR product and pACYCduet plasmid were PCR cleaned up with the same cleanup kit stated above, and the digested *tacT* product was ligated into MCS1 of digested pACYCDuet with T4 DNA ligase (Fisher) at room temperature for 30 min. Once transformants were verified, the *tacA* PCR product was digested with EcoRI and XhoI for 1 h at 37°C, PCR cleaned, and ligated as described above into the second MCS of pACYCDuet, which had the *tacT* gene cloned into MCS1.

Plasmids were isolated using the Wizard Plus SV miniprep kit (Promega). To confirm the correct sequences were cloned without mutations, DNA sequencing reactions were analyzed at the Georgia Genomics Facility, University of Georgia—Athens. Site-directed mutagenesis (Stratagene) was performed on pTacAT-2 or pTacAT-1 to change the mentioned residues K12, K44, and K83 to A, Q, or R. PCR was performed using Pfu Ultra II DNA polymerase with the primers listed in Table S2. Modifications included an annealing time of 60 s, an extension temperature of 68°C, and an extension time of 2.5 min kb^−1^. DNA changes were confirmed by sequencing.

### Protein purification.

Plasmids encoding H_6_-TacT^WT^ and TacA^WT^ (pTacAT-2), H_6_-TacT^WT^ and TacA^K12A^ (pTacAT-12), H_6_-TacT^WT^ and TacA^K44A^ (pTacAT-13), H_6_-TacT^WT^ and TacA^K44Q^ (pTacAT-16), H_6_-TacT^WT^ and TacA^K44R^ (pTacAT-15), and His_6_-TacT^WT^ and TacA^K83A^ (pTacAT-14) were electroporated into the Δ*pat* variant of *Escherichia coli* strain C41(λDE3) (42) to create strain JE9314. Cultures of cells containing plasmids were grown to stationary phase (OD_650_ of ~1.3) and subcultured (1:100 [vol/vol]) into 6 liters of LB plus chloramphenicol. Cultures were grown with shaking at 25°C to an OD_650_ of 0.5, after which ectopic gene expression was induced with IPTG (isopropyl-β-d-thiogalactopyranoside [0.5 mM]). Cultures were grown overnight at 25°C, cells were harvested by centrifugation at 6,000 × *g* for 15 min at 4°C, and cell pellets were stored at −80°C until used.

Cell pellets were resuspended in 50 ml of buffer A containing 4-(2-hydroxymethyl)-1-piperazineethanesulfonic acid (50 mM HEPES [pH 7.0 at 4°C]), NaCl (500 mM), imidazole (20 mM), glycerol (20% [vol/vol]), lysozyme (1 mg/ml), DNase (1 μg/ml), and protease inhibitor phenylmethylsulfonyl fluoride (PMSF [1 mM]). Cells were sonicated for 60 s using a Qsonica sonicator at 60% duty with 2-s pulses. Lysates were centrifuged using a Beckman Coulter, Inc., Avanti J-251 centrifuge equipped with a JA-25.50 rotor at 40,000 × *g* for 30 min. Clarified lysates were filtered through a 0.45-μm-pore filter and applied to a 2-ml HisTrap FF column (GE Healthcare Sciences) using an Äkta fast protein liquid chromatography (FPLC) system (GE Healthcare Sciences). The column was washed with 10 column volumes of bind buffer, 7 column volumes of 8% elution buffer (50 mM HEPES [pH 7.0 at 4°C], 500 mM NaCl, 500 mM imidazole, 20% [vol/vol] glycerol), and a 20-column-volume gradient to 100% elution buffer. When separation of TacT and TacA complexes was necessary, proteins were denatured on the column as described previously (31). Fractions were run on an SDS-PAGE gel, and fractions containing the desired protein were combined and dialyzed for 3 h each in storage buffer 1 (50 mM HEPES [pH 7.0 at 4°C], 400 mM NaCl, 20% [vol/vol] glycerol), storage buffer 2 (50 mM HEPES [pH 7.0 at 4°C], 200 mM NaCl, 20% [vol/vol] glycerol), and storage buffer 3 (50 mM HEPES [pH 7.0 at 4°C], 150 mM NaCl, 20% [vol/vol] glycerol). Proteins were flash frozen in liquid N_2_ and stored at −80°C. Proteins were quantified using a NanoDrop 1000 spectrophotometer (Thermo Scientific) using the molecular mass (28.67 kDa) and extinction coefficient (18,700 M^−1^ cm^−1^ [Expasy ProtParam]) of the complex (assuming a 1:1 ratio of TacT to TacA, as determined via size exclusion chromatography [described below]). Percentage of purity was calculated using ImageQuant v5.2 software. CobB protein was purified as described elsewhere (43). TacT was isolated from TacAT complex using a purification protocol reported elsewhere (31). Briefly, TacAT^WT^ complex was purified as described above and was separated into its components by denaturation with guanidine-HCl (5 M) followed by overnight dialysis. Denatured proteins were resolved by Ni-affinity chromatography and refolded by dialysis of the denaturant. As refolding occurred, the bulk of TacT became insoluble, while TacA remained stable in solution. This procedure yielded 6-fold larger amounts of TacA than TacT (e.g., 0.5 mg of TacA versus 0.08 mg TacT per liter of culture).

### Size exclusion chromatography.

A Superose 12 10/300 GL gel filtration column (GE Healthcare Life Sciences) was equilibrated as per the manufacturer’s protocol using water and elution buffer (50 mM HEPES [pH 7.0 at 4°C], 150 mM NaCl, 20% [vol/vol] glycerol). Samples were applied to the column using a 100-μl superloop. Gel filtration standards (Bio-Rad) were applied to the column first, and a standard curve was calculated using the log molecular weight (MW_log_) of each standard against the retention time of each protein. Purified TacAT complexes were eluted from the column in the same manner, retention times were recorded, and molecular weights were determined using an equation calculated from the standard curve.

### *In vitro* acetylation assays.

Homogeneous TacAT complex (3 μM) was incubated with or without [1-^14^C]acetyl-CoA (20 μM) in HEPES (50 mM [pH 7.5]) and *tris-*(2-carboxyethyl)phosphine (TCEP [1 mM]) for 1 h at 37°C in a total volume of 25 μl. Reactions were quenched by the addition of SDS loading buffer (60% [vol/vol] glycerol, 0.3 M Tris-HCl [pH 6.8], 12 mM EDTA, 12% SDS, 0.87 mM 2-mercaptoethanol, 0.05% [wt/vol] bromophenol blue), and reaction mixtures were resolved by SDS-PAGE on a 15% (wt/vol) polyacrylamide gel with Tris-HCl buffer at pH 8.8 for the resolving gel or Tris-HCl at pH 6.8 for the stacking gel. Samples were run at 200 V for 45 min. Transfer of the radiolabel onto TacA was visualized using a Typhoon Trio Plus variable mode imager (GE Healthcare).

### *In vitro* deacetylation assays.

To determine whether or not TacA^Ac^ was a substrate for CobB sirtuin, 200-μl reaction mixtures containing radiolabeled TacA^Ac^ synthesized as described above were treated with NAD^+^-dependent CobB sirtuin deacetylase [1-^14^C]acetyl-CoA. Briefly, excess [1-^14^C]acetyl-CoA was removed by buffer exchange using AmiconUltra 0.5-ml centrifugal filters (Ultracell, 10,000 molecular weight cutoff [MWCO]) and HEPES buffer (50 mM [pH 7.5]). Reaction mixtures were concentrated to 100 μl and served as a 2× stock of TacA^Ac^. After removal of [1-^14^C]acetyl-CoA, TacA^Ac^ was added to reaction mixtures (1×) that contained (i) NAD^+^ (1 mM) plus CobB (75 pmol; 3 μM final concentration), (ii) NAD^+^ (1 mM) plus CobB (3 μM), or (iii) NAD^+^ (1 mM) plus CobB (3 μM) plus nicotinamide (5 mM). Samples were incubated at 37°C for 1 h and then resolved by SDS-PAGE. Deacetylation of TacA^Ac^ was monitored with a phosphorimager as described above.

### *In vitro* DHFR protein synthesis assay.

The PureExpress *in vitro* protein synthesis kit (New England Biolabs) reactions were set up per the manufacturer’s protocol in RNase-free tubes with the following modifications. Reaction mixtures were adjusted to a total volume of 15 μl (5 μl of solution A, 3.75 μl solution B, 100 ng DHFR DNA) and, when noted, supplemented with acetyl-CoA (2 mM), TacAT complex (2 μM), or TacT (2 μM). All samples contained 0.5 μl of SUPERase In RNase inhibitor (Thermo, Fisher Scientific). Reaction mixtures were brought up to equal volumes with the addition of RNase-free water, per the manufacturer’s protocol. Reaction mixtures were incubated in a 37°C sand bath for 2 h, after which tubes were placed on ice, and 2.5 μl of each reaction mixture was added to 12 μl of 1× loading dye (60% [vol/vol] glycerol, 0.3 M Tris-HCl [pH 6.8], 12 mM EDTA, 12% SDS, 0.87 mM 2-mercaptoethanol, 0.05% [wt/vol] bromophenol blue) and heated at 100°C for 10 min. Five microliters of each denatured sample was resolved by SDS-PAGE on a 15-lane 15% (wt/vol) polyacrylamide gel with Tris-HCl buffer at pH 8.8 for the resolving gel or Tris-HCl at pH 6.8 for the stacking gel. Gels were run for 45 min at 220 V and visualized via Coomassie blue staining and acetic acid destaining. Gels were imaged and analyzed for DHFR protein production using ImageQuant v5.2 software. The intensity of DHFR from each lane on the polyacrylamide gel was normalized to the asterisk-indicated bands in Fig. 3 using the ImageQuant v5.2 software. These values were used to calculate the percentage of decrease in DHFR per reaction. The mean percentages were plotted using Prism6 software to obtain the standard deviation of each percentage of decrease as shown at the bottom of the figure.

### DNA-binding assays.

Electrophoretic mobility shift DNA-binding assays were performed using DNA probes with 6-carboxyfluorescein (6-FAM) covalently attached to the 5′ end of the probe. The primers used in these experiments were manufactured by Integrated DNA Technologies, Inc. (IDT [Coralville, IA]). Probes were generated from PCR amplification of strain JE10079 (*tacAT*^+^) chromosomal DNA. PCR products were sized on a 1% (wt/vol) agarose gel and were purified using a Wizard SV gel and the PCR cleanup system (Promega). Binding reaction mixtures contained 6-FAM double-stranded DNA (dsDNA) probe (50 ng), HEPES-NaOH buffer (50 mM [pH 7]) containing KCl (50 mM), MgCl_2_ (10 mM), disodium ethylenetetraacetic acid (Na_2_EDTA [0.5 mM]), glycerol (10% [vol/vol]), 25 μg/μl poly(dI-dC) (Sigma-Aldrich), and, when added, TacT-TacA complex protein in molar excess of probe, as indicated in the figure legends. Reaction mixtures (25 μl) were incubated at 25°C for 45 min. Glycerol (27 μmol; 5 μl of a 50% [vol/vol] solution) was added to reaction mixtures, which were resolved using a nondenaturing Criterion Tris-HCl buffer (375 mM [pH 8.6]) and 7.5% (wt/vol) polyacrylamide gel (Bio-Rad) at 120 V. Gels were imaged using a Typhoon Trio Plus variable mode imager (GE Healthcare) at wavelength 488 nm (blue) and analyzed with ImageQuant v5.2 software.

### Construction of a strain carrying a chromosomal *tacA* allele encoding TacA^K44Q^ or TacA^K44R^.

A region 792 bp upstream of *tacAT* in frame with the coding region of *tacAT* was cloned into pCV1 (pTacAT-17). Site-directed mutagenesis was performed to mutate *tacA* to encode either TacA^K44R^ or TacA^K44Q^, as described above (pTacAT-22 and pTacAT-23). This DNA fragment (792 bp upstream of *tacAT* in frame with *tacAT* coding for variants) was designated fragment 1 (Fig. S8). Gene splicing by overlap extension quantitative PCR (SOE-qPCR) was utilized to fuse this fragment to a *cat*^*+*^ gene (fragment 2) and the downstream region of *tacAT* (fragment 3). The *cat*^*+*^ gene from pKD3 (37) was used as a template for fragment 2. Fragments 1, 2, and 3 were amplified with Pfu Ultra II fusion DNA polymerase (Stratagene) with primers listed in Table S2 and an annealing temperature of 61.8°C. PCR products were sized on a 1% (wt/vol) agarose gel and were purified using a Wizard SV PCR cleanup system (Promega). Fragments 1 and 2 (50 ng each per 50-μl PCR mixture) were annealed using SOE-PCR primers (5′ SOE-PCR fragment 1 and 3′ SOE-PCR fragment 2 [Table S2]) with an annealing temperature of 61.8°C and an extension time of 30 s/kb. PCR products were sized on a 1% (wt/vol) agarose gel and were purified using a Wizard SV PCR cleanup system (Promega). Fragments 1 and 2 were annealed to fragment 3 using the same protocol. The linear PCR fragments 1 to 3 were transformed into a Δ*tacAT* strain harboring the helper plasmid pKD46 using the protocol described above under “Strain construction” (37). The linear PCR fragment recombined with the upstream and downstream regions of *tacAT*, which inserted the *tacAT* operon coding for K44Q or K44R in place of its absence. Cells were plated on LB agar plus chloramphenicol (10 μg/ml), and individual colonies were screened for acquisition of *tacAT* compared to Δ*tacAT*. Chromosomal mutations were confirmed by sequencing.

### RNA isolation.

Strains JE10079 (*tacAT*^+^), JE23754 (*tacA1* encoding TacA^K44Q^T^WT^), JE23755 (*tacA2* encoding TacA^K44R^T^WT^), and JE23438 (Δ*tacT*) were grown overnight in triplicate in nutrient broth (NB [2 ml]; Difco) with shaking at 37°C. After incubation, strains were diluted 1:100 into 5 ml of fresh NCE minimal medium supplemented with MgSO_4_ (1 mM), Wolfe’s trace minerals (1×), and glycerol (22 mM). Cultures were grown with shaking at 37°C to an optical density at 600 nm of 0.5, and then 5 ml of each sample were quickly centrifuged in 1.5-ml Eppendorf tubes at 6,000 × *g*, supernatant was removed, and pellets were flash-frozen in liquid nitrogen and kept on dry ice. RNA was isolated following the RNAsnap protocol (33). Pellets were resuspended in 150 μl of boil solution (18 mM EDTA, 0.025% [wt/vol] SDS, 95% [vol/vol] RNA-grade formamide, 1% [vol/vol] 2-mercaptoethanol in RNase-free water) and were mixed vigorously to break up the cell pellet. Pellets were incubated at 95°C for 7 min and centrifuged at 16,000 × *g* for 5 min at room temperature; 100 μl of supernatant was transferred to a fresh tube. A sodium acetate-ethanol RNA precipitation was then conducted by the addition of 300 μl of RNase-free water, 40 μl of sodium acetate (3 M [pH 5.2]; final concentration, 0.3 M), and finally 400 μl of ice-cold absolute ethanol (100%), with mixing briefly before the addition of the next reagent. The mixture was incubated on ice for 15 min and centrifuged at 16,000 × *g* for 15 min at 4°C, and ethanol was decanted off. Ethanol (400 μl, cold, 70% [vol/vol]) was added, and pellets were centrifuged at 12,000 × *g* for 10 min at 4°C in an Eppendorf 5415D centrifuge. Ethanol was removed, and pellets were allowed to dry. RNA pellets were resuspended in RNase-free water at 4°C on ice overnight. Subsequent RNase-free DNase I treatment was conducted using the Ambion Turbo DNA-free kit according to the manufacturer’s instructions (Thermo, Fisher Scientific). After DNA cleavage, a final sodium acetate-ethanol precipitation was performed as described above. RNA was allowed to resuspend at 4°C on ice for 4 h, then was flash-frozen in liquid nitrogen and stored at −80°C until used. A small aliquot of each sample was sent for quality control analysis using the RNA 600 Nano kit of the Agilent 2100 bioanalyzer through the Georgia Genomics Facility. Primers for qPCR were designed using primer 3 software and were evaluated for specificity and melting curve prior to running the qPCR.

### cDNA synthesis and quantitative reverse transcription PCR.

Total RNA (620 ng) from each sample was used for the synthesis of cDNA using the iScript cDNA synthesis kit from Bio-Rad Laboratories according to the manufacturer’s protocol. Each cDNA reaction mixture was then diluted to 7.5 ng/μl and used as the template for PCR. For real-time PCR, 20-μl reaction mixtures were prepared with 10 μl of 2× FastSYBR green master mix (Applied Biosystems), 500 nM each gene-specific primer (1 μl of 10 μM primer stock), and 15 ng of cDNA (2 μl of 7.5-ng/μl cDNA). The real-time PCR was performed using a 7500 Fast real-time PCR system (Applied Biosystems). The threshold cycle values of *rpoB* and *gyrB* were checked first to ensure that both genes were optimal for use as reference genes for these strains under the conditions chosen for RT-qPCR. Cycle threshold (*C*_*T*_) data were normalized to the *rpoB* gene (44). These normalized values (Δ*C*_*T*_) were transformed using 2(e−Δ*C*_*T*_)/10^−6^ (45) and were reported as arbitrary gene expression units (EU), or the gene expression ratio of the mutant strains to the parent strain (JE10079 *tacAT*^+^). Mean EU values were used to calculate the standard error of the mean (SEM) using Prism6 from three biological replicates that were each tested in technical triplicates. Differences in EU between mutant strains and JE10079 and between JE23754 (*tacA1* encoding TacA^K44Q^T) and JE23755 (*tacA2* encoding TacA^K44R^T) were compared using Welch’s *t* test with the GraphPad Prism6 software as shown in Fig. 6.

### Reagent and resource sharing.

Further information and requests for reagents may be directed to and will be fulfilled by the corresponding author, Jorge C. Escalante-Semerena.
