# Supplementary figures and images for: A Toxin Involved in Salmonella Persistence Regulates Its Activity by Acetylating Its Cognate Antitoxin, a Modification Reversed by CobB Sirtuin Deacetylase
Source: mBio. 2017 May 30;8(3):e00708-17. doi: 10.1128/mBio.00708-17 (PMC5449658; doi:10.1128/mBio.00708-17)

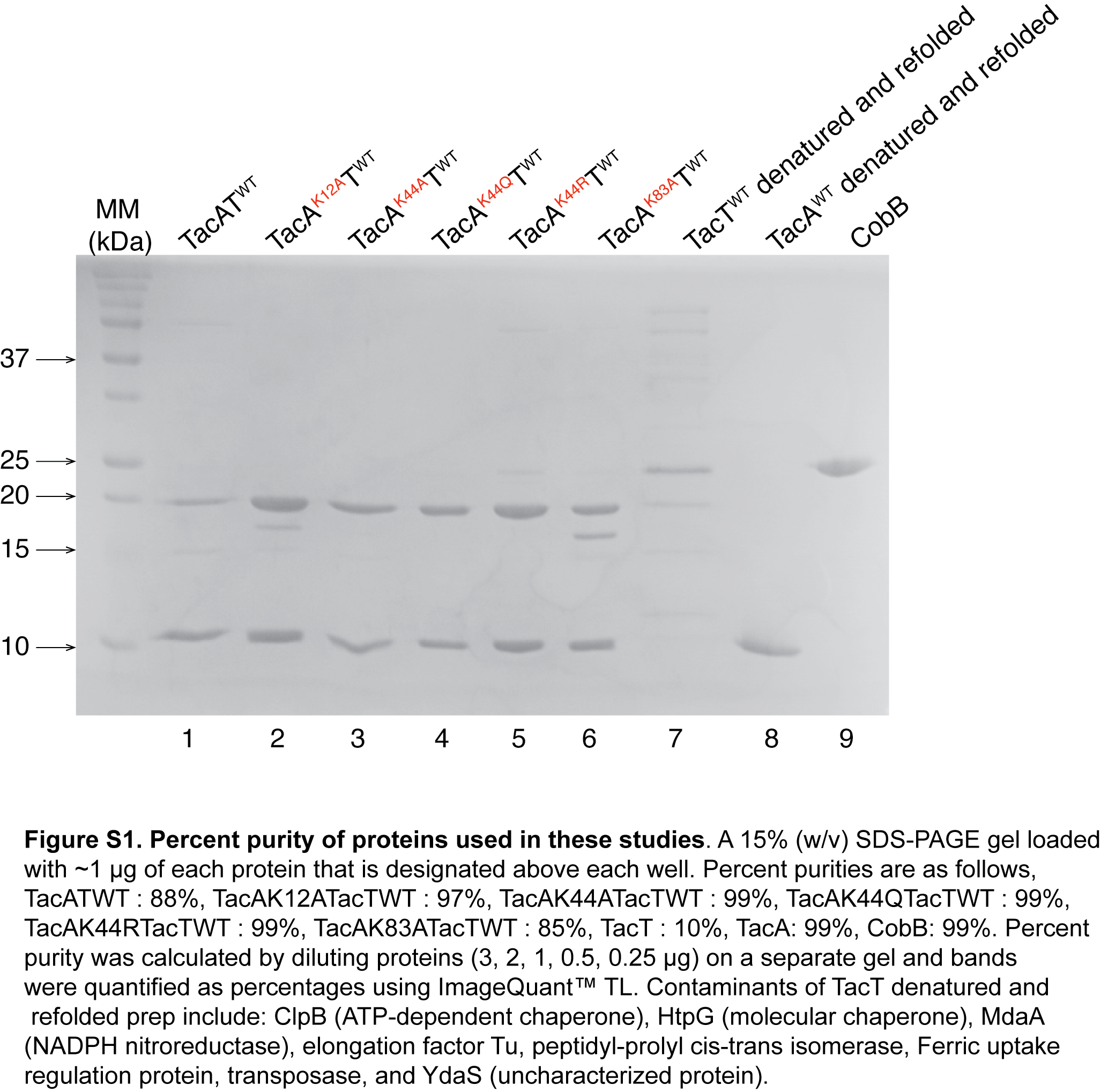

Supplement: FIG S1 [file mbo003173326sf1.tif]

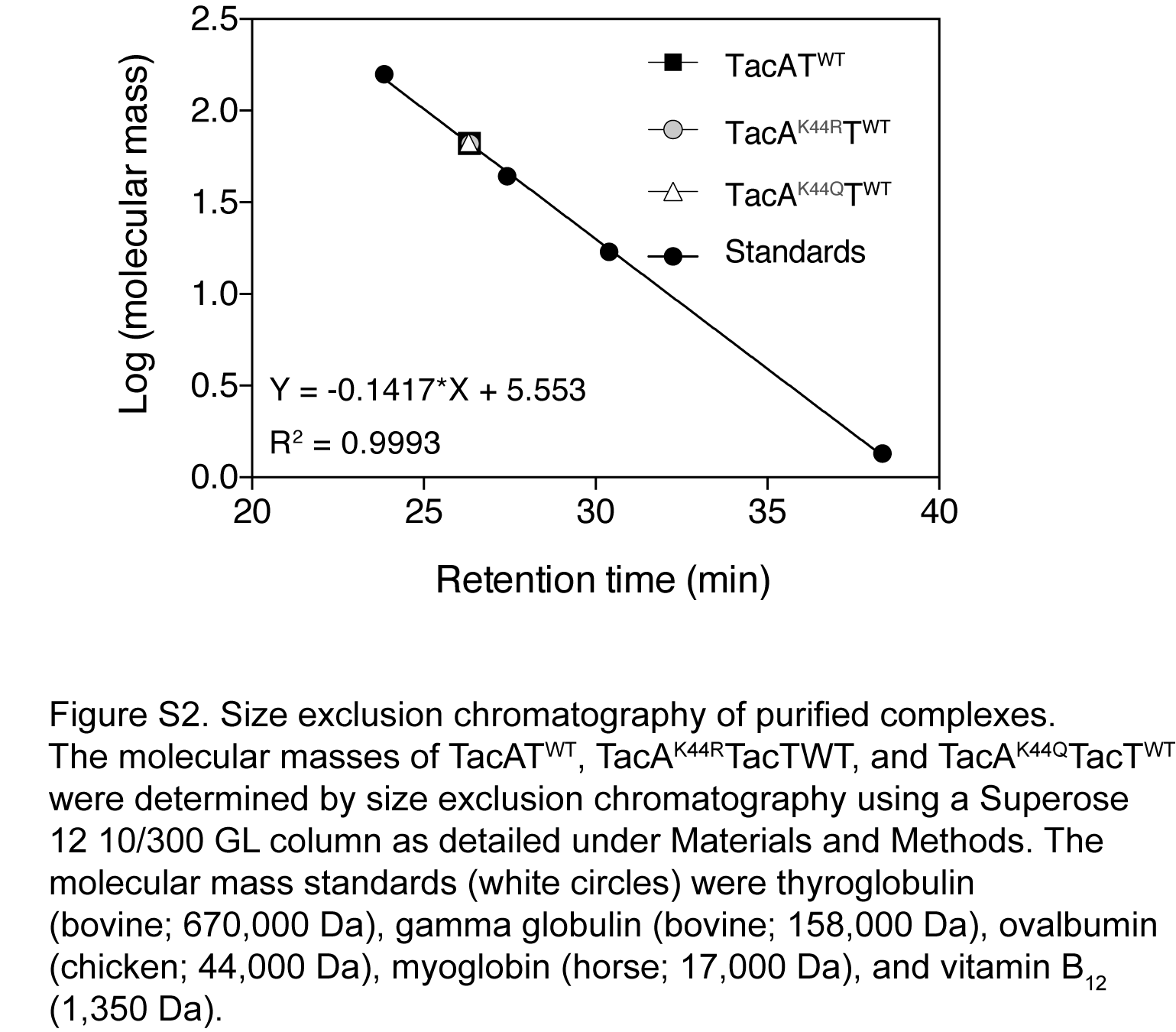

Supplement: FIG S2 [file mbo003173326sf2.tif]

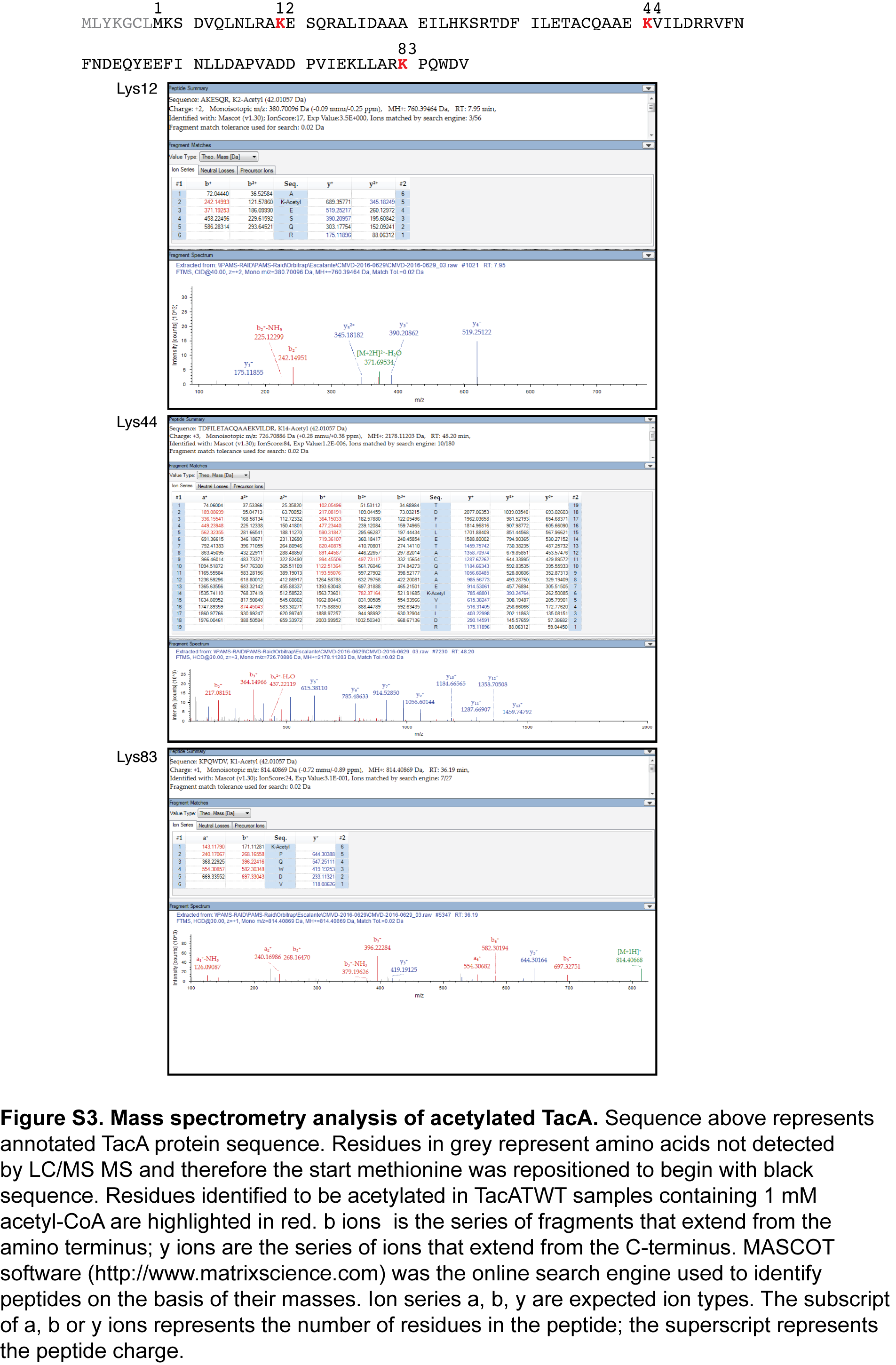

Supplement: FIG S3 [file mbo003173326sf3.tif]

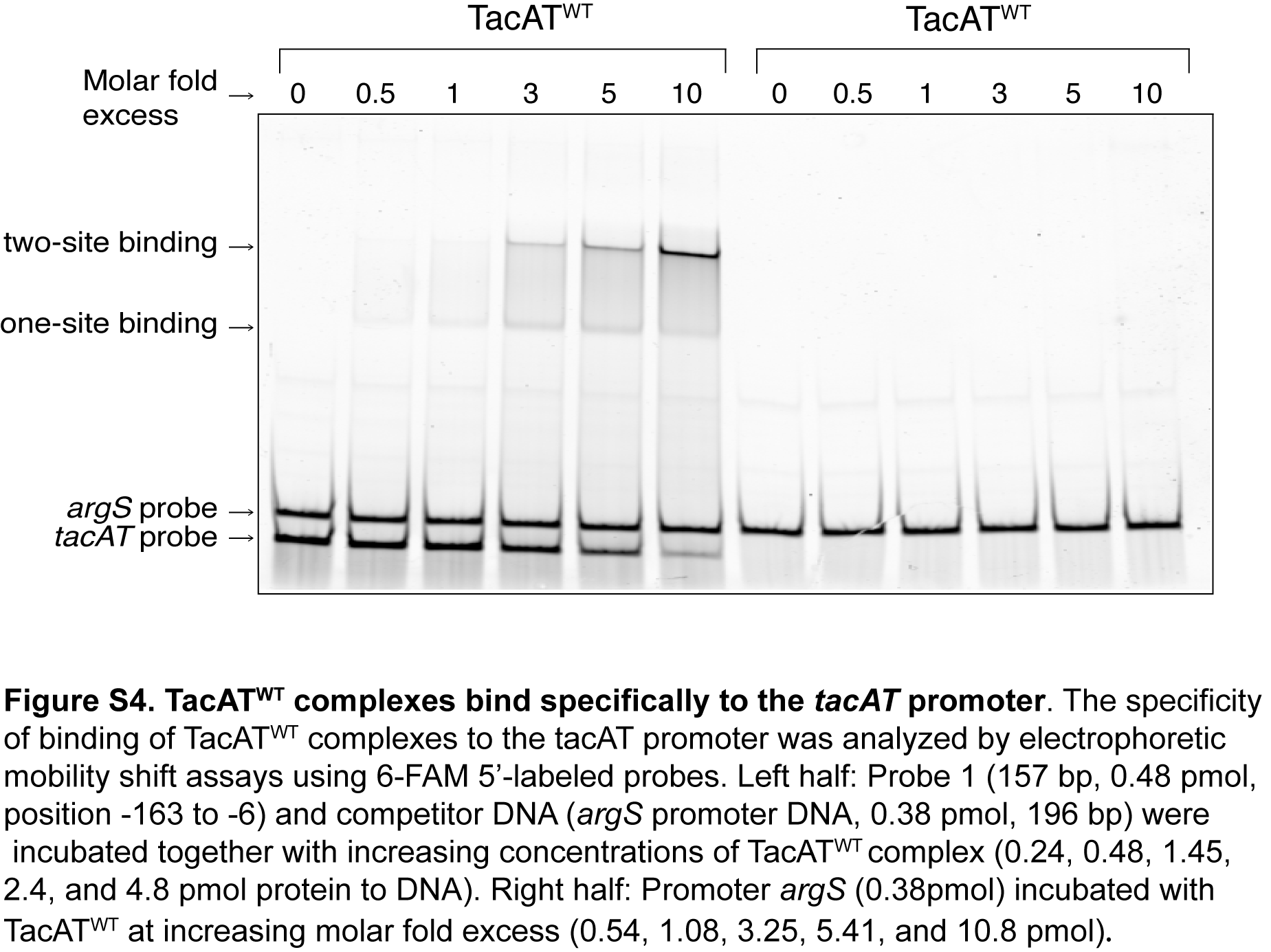

Supplement: FIG S4 [file mbo003173326sf4.tif]

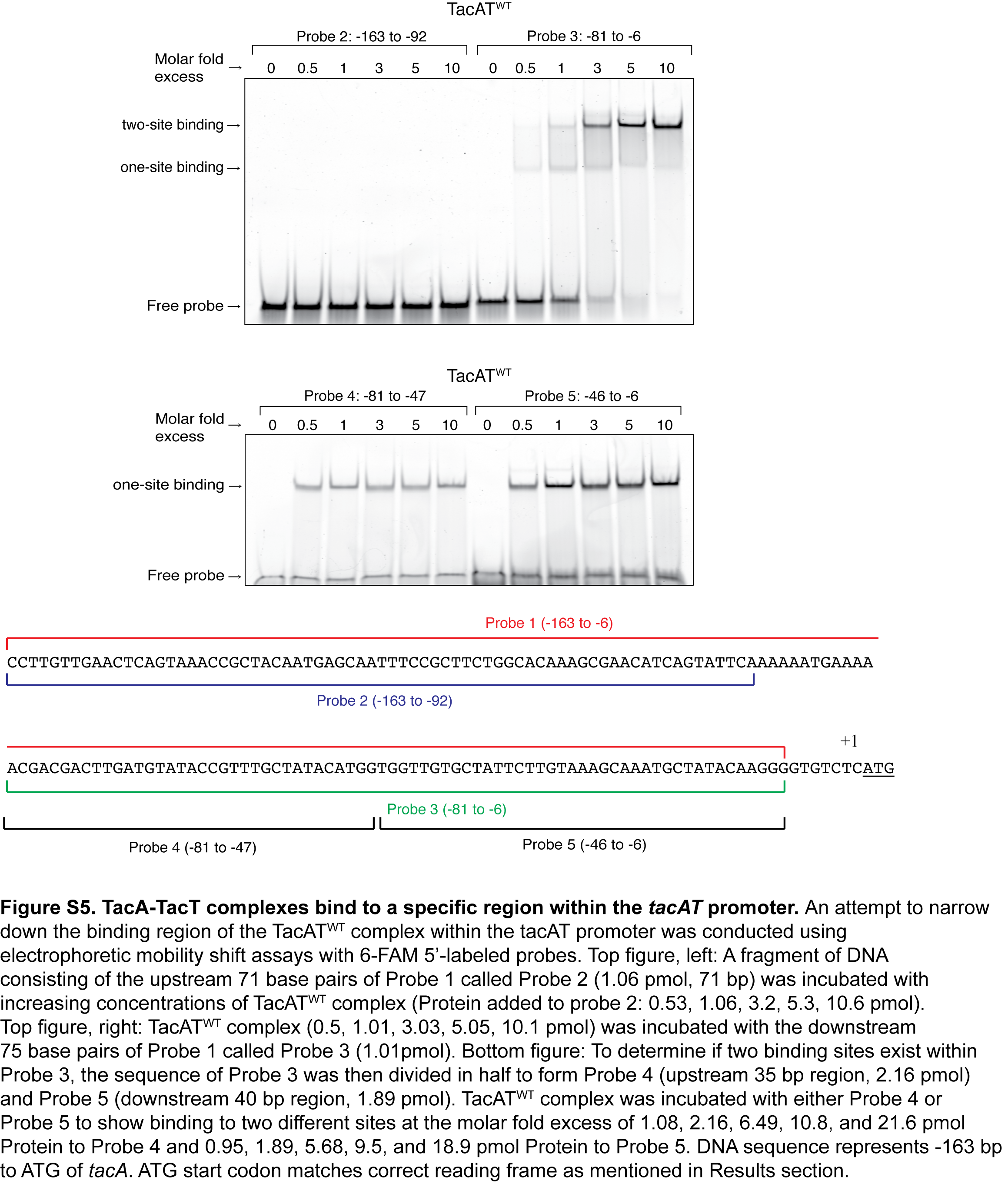

Supplement: FIG S5 [file mbo003173326sf5.tif]

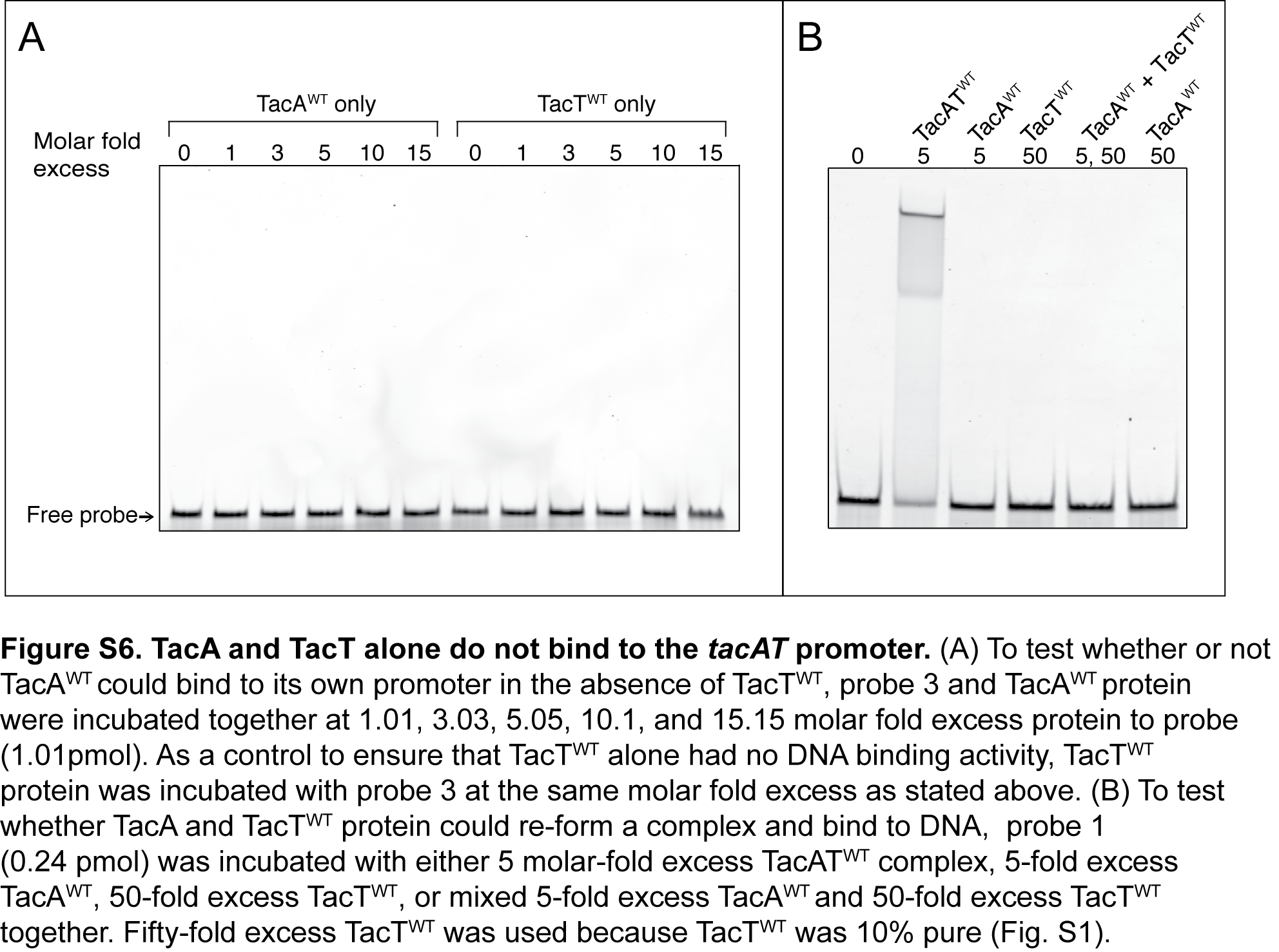

Supplement: FIG S6 [file mbo003173326sf6.tif]

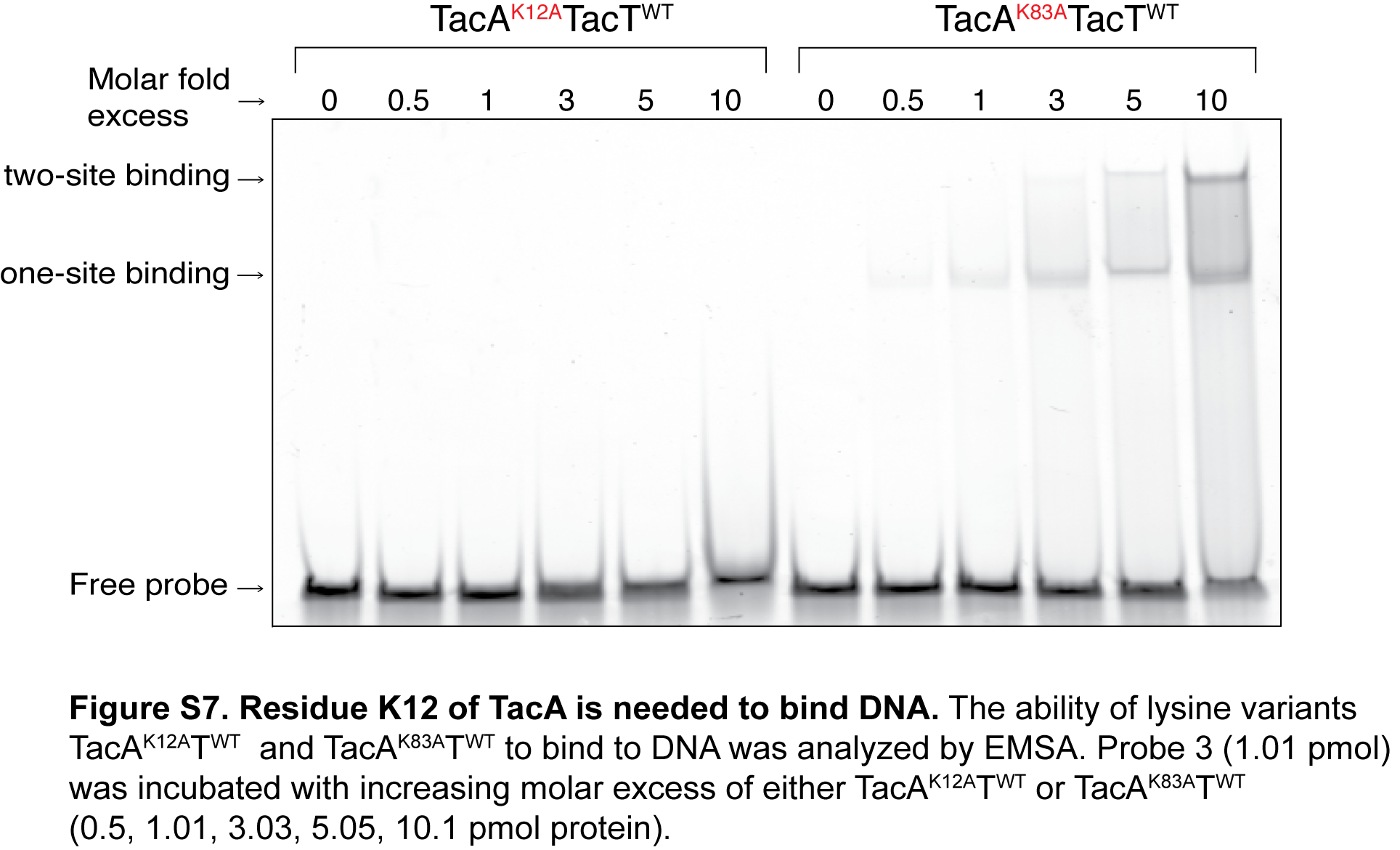

Supplement: FIG S7 [file mbo003173326sf7.tif]

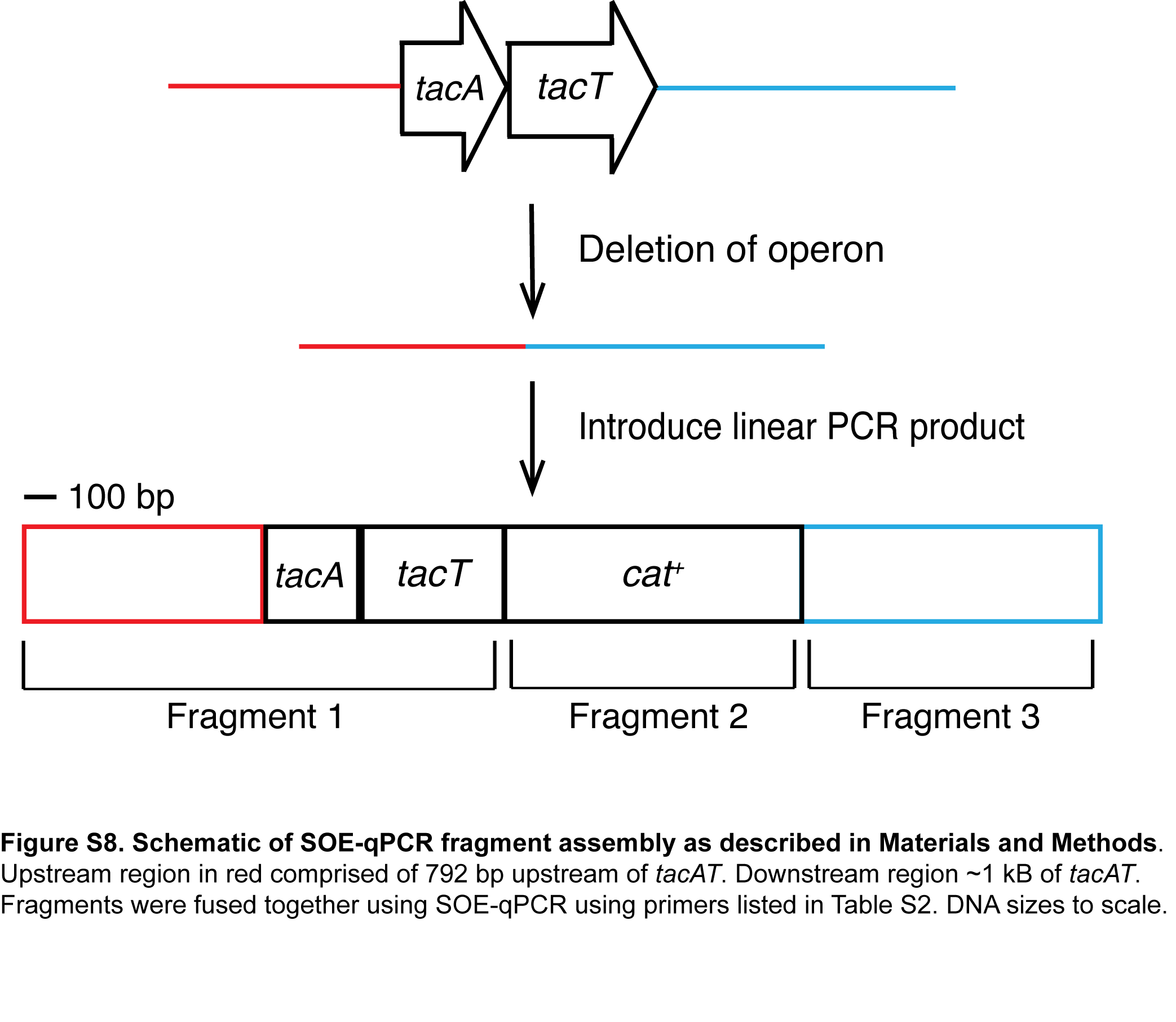

Supplement: FIG S8 [file mbo003173326sf8.tif]
