# Supplementary material for: A Toxin Involved in Salmonella Persistence Regulates Its Activity by Acetylating Its Cognate Antitoxin, a Modification Reversed by CobB Sirtuin Deacetylase
Source: mBio. 2017 May 30;8(3):e00708-17. doi: 10.1128/mBio.00708-17 (PMC5449658; doi:10.1128/mBio.00708-17)
Supplement: TABLE S1 [file mbo003173326st1.docx]

| **Table S1. Strains and plasmids used in this work** | | |
| --- | --- | --- |
| ***S. enterica* strains^a^** | **Relevant genotype** | **Description; Source^b^, Reference** |
| JE10079 | *ara-9* | Laboratory collection |
| **Derivatives of JE10079** |  |  |
| JE21469 | *tacAT^+^* / pCV1 |  |
| JE21486 | *tacAT^+^* / pTacT-4 |  |
| JE21493 | *tacA::cat^+^* / pCV1 |  |
| JE21494 | *tacA::cat^+^* / pTacT-4 |  |
| JE21489 | *tacT::cat^+^*/ pCV1 |  |
| JE21490 | *tacT::cat^+^*/ pTacT-4 |  |
| JE21500 | *tacAtacT::cat^+^*/ pCV1 |  |
| JE21501 | *tacAtacT::cat^+^*/ pTacT-4 |  |
| JE21503 | *tacAtacT::cat^+^*/ pTacAT-1 |  |
| JE23697 | *tacAtacT::cat^+^*/ pTacA^K44R^T |  |
| JE23698 | *tacAtacT::cat^+^*/ pTacA^K44Q^T |  |
| JE23754 | *tacA1 tacT^+^* |  |
| JE23755 | *tacA2 tacT^+^* |  |
| JE23438 | Δ*tacT* | Laboratory collection |
| JE10813 | Δ*ara-9* / pKD46 | Laboratory collection |
| ***E. coli* strains** |  |  |
| *E. coli* C41 (λDE3) | *pka12*::*kan^+^ ompT hsdS (r_B_m_B_) gal λ (DE3)* | Laboratory collection |
| *E. coli* DH5α | Φ80d*lacZ* ΔM15 *recA*1 *endA*1 *gyrA*96 *thi*-1 *hsdR*17 (r_k_^-^, m_k_^+^) *supE*44 *relA*1 *deoR* Δ(*lacZYA-argF)* U169 *phoA* | NEB |
| **Plasmid** | **Genotype** | **Description; Source, Reference** |
| pCV1 | *araC^+^ bla^+^* | pBAD24 with BspQI MCS  (1) |
| pCV3 | *araC^+^ cat^+^* | pBAD33-SD1 with BspQI MCS  (1) |
| pACYCDuet-1 | *cat^+^* | Vector for coexpression of two genes; Novagen |
| pTacT-4 | *tacT^+^ bla^+^* | *tacT^+^* cloned into pCV1 |
| pTacAT-1 | *tacAT^+^ bla^+^* | *tacAT^+^* cloned into pCV1 |
| pTacAT-2 | *tacAT^+^ cat^+^* | pACYCDuet plasmid with *tacT* cloned into MCS1, *tacA* cloned into MCS2 |
| pTacAT-12 | *tacAT^+^ cat^+^* | pACYCDuet plasmid with *tacT* cloned into MCS1, *tacA* coding for TacA^K12A^ cloned into MCS2 |
| pTacAT-13 | *tacAT^+^ cat^+^* | pACYCDuet plasmid with *tacT* cloned into MCS1, *tacA* coding for TacA^K44A^ cloned into MCS2 |
| pTacAT-14 | *tacAT^+^ cat^+^* | pACYCDuet plasmid with *tacT* cloned into MCS1, *tacA* coding for TacA^K83A^ cloned into MCS2 |

| pTacAT-15 | *tacAT^+^ cat^+^* | pACYCDuet plasmid with *tacT* cloned into MCS1, *tacA* coding for TacA^K44R^ cloned into MCS2 |
| --- | --- | --- |
| pTacAT-16 | *tacAT^+^ cat^+^* | pACYCDuet plasmid with *tacT* cloned into MCS1, *tacA* coding for TacA^K44Q^ cloned into MCS2 |
| pTacAT-9 | *tacAT^+^ bla^+^* | *tacAT^+^* cloned into pCV1, *tacA* coding for TacA^K44R^ |
| pTacAT-10 | *tacAT^+^ bla^+^* | *tacAT^+^* cloned into pCV1, *tacA* coding for TacA^K44Q^ |
| pTacAT-17 | *P_tacAT_ bla^+^* | Upstream region (-792 bp) of *tacAT^+^* with *tacAT^+^* cloned into pCV1 |
| pTacAT-22 | *tacAT^+^ bla^+^* | Upstream region (-792 bp) of *tacAT^+^* with *tacAT* (coding for TacA^K44R^) cloned into pCV1 |
| pTacAT-23 | *P_tacAT_ bla^+^* | Upstream region (-792 bp) of *tacAT^+^* with *tacAT* (coding for TacA^K44Q^) cloned into pCV1 |
| pCobB71 | *cobB^+^ bla^+^* | *cobB* ORF in pTEV6;  (2) |
| pKD46 | *bla^+^* | Express the Red system to avoid unwanted recombination;  (3) |
| pKD3 | *cat^+^* | Template plasmid carrying *cat^+^* gene; (3) |

a All *Salmonella* strains used were derivatives of *S. enterica* subsp. *enterica* sv. Typhimurium strain LT2.

^b^ Unless otherwise stated all strains and plasmids were engineered during the course of this work

**REFERENCES**

1. VanDrisse CM, Escalante-Semerena JC. 2016. New high-cloning-efficiency vectors for complementation studies and recombinant protein overproduction in Escherichia coli and Salmonella enterica. Plasmid doi:10.1016/j.plasmid.2016.05.001.

2. Tucker AC, Escalante-Semerena JC. 2010. Biologically active isoforms of CobB sirtuin deacetylase in *Salmonella enterica* and *Erwinia amylovora*. J Bacteriol 192:6200-6208.

3. Datsenko KA, Wanner BL. 2000. One-step inactivation of chromosomal genes in *Escherichia coli* K-12 using PCR products. Proc Natl Acad Sci USA 97:6640-6645.
