# Supplementary material for: A Toxin Involved in Salmonella Persistence Regulates Its Activity by Acetylating Its Cognate Antitoxin, a Modification Reversed by CobB Sirtuin Deacetylase
Source: mBio. 2017 May 30;8(3):e00708-17. doi: 10.1128/mBio.00708-17 (PMC5449658; doi:10.1128/mBio.00708-17)
Supplement: TABLE S2 [file mbo003173326st2.docx]

| **Table S2. Primers and DNA probes used in this study.** | |
| --- | --- |
| **Primer Name** | **Primer Sequence 5’ 🡪 3’** |
| **Strain construction** | |
| 5’ *tacT* Wanner primer | CACCAGAACCTTTGTCCGCTTTTCATCAGGTAGCTGGTGTAGGCTGGAGCTGCTTC |
| 3’ *tacT* Wanner primer | CGCTTATAGCGATTTGAACATAACAGTCTTTGCATTCTATTGAGGGAGCCCATATGAATATCCTCCTTAG |
| 5’ *tacAT* Wanner primer | GCTATACATGGTGGTTGTGCTATTCTTGTAAAGCAAATGGTGTAGGCTGGAGCTGCTTC |
| 3’ *tacAT* Wanner primer | CGCTTATAGCGATTTGAACATAACAGTCTTTGCATTCTATTGAGGGAGCCCATATGAATATCCTCCTTAG |
| 5’ *tacA* Wanner primer | GCTATACATGGTGGTTGTGCTATTCTTGTAAAGCAAATGGTGTAGGCTGGAGCTGCTTC |
| 3’ *tacA* Wanner primer | GGTTTCCTTGCCAGCAGTTTTTCGATAACGGGATCATCTGCGACCGGTGCCATATGAATATCCTCCTTAG |
| 5’ SOE PCR fragment 1 | AATTAACAAGGTCTGTACAGGGATGCTATCAG |
| 3’ SOE PCR fragment 1 | CTCCAGCCCTCCAGCCTACACCTATTGAGGGAGCCTAAGGAACAATGT |
| 5’ SOE PCR fragment 2 | CTCCCTCAATAGGTGTAGGCTGGAGCTGCT |
| 3’ SOE PCR fragment 2 | CCGGCTGTCAGGGCATATGAATATCCTCCTTAG |
| 5’ SOE PCR fragment 3 | GATATTCATATGCCCTGACAGCCGGAAAGG |
| 3’ SOE PCR fragment 3 | CGAAACGCGAAAAACCCACG |
| **Site-Directed Mutagenesis** | |
| 5’ TacA^K12A^ | GCCCGCTGCGATTCCGCAGCTCTAAGGTTAAGTTGAAC |
| 3’ TacA^K12A^ | GTTCAACTTAACCTTAGAGCTGCGGAATCGCAGCGGGC |
| 5’ TacA^K44A^ | TGCCAGGCTGCCGAGGCAGTGATCCTTGACCG |
| 3’ TacA^K44A^ | CGGTCAAGGATCACTGCCTCGGCAGCCTGGCA |
| 5’ TacA^K83A^ | CTGCTGGCAAGGGCGCCTCAGTGGGACGTG |
| 3’ TacA^K83A^ | CACGTCCCACTGAGGCGCCCTTGCCAGCAG |
| 5’ TacA^K44R^ | GTCAAGGATCACTCTCTCGGCAGCCTGGCAG |
| 3’ TacA^K44R^ | CTGCCAGGCTGCCGAGAGAGTGATCCTTGAC |
| 5’ TacA^K44Q^ | CGGTCAAGGATCACCTGCTCGGCAGCCTGGC |
| 3’ TacA^K44Q^ | GCCAGGCTGCCGAGCAGGTGATCCTTGACCG |
| **Cloning ^b^** | |
| 5’ *tacT* cloned into pCV1 | NNGCTCTTCNTTCGTGGGACGTGTAACAGCACC |
| 3’ *tacT* cloned into pCV1 | NNGCTCTTCNTTACTATTGAGGGAGCCTAAGGA |
| 5’ *tacA* cloned into pCV1 | NNGCTCTTCNTTCATGCTATACAAGGGGTGTCT |
| 3’ *tacA* cloned into pCV1 | NNGCTCTTCNTTATTACACGTCCCACTGAGGTT |
| 5’ *tacT* cloned into MCS1 pACYCDuet BamHI | NNNNNNGGATCCGTGGGACGTGTAACA |
| 3’ *tacT* cloned into MCS1 pACYCDuet EcoRI | NNNNNNGAATTCCTATTGAGGGAGCCTAAGGAACAATGTTC |
| 5’ *tacA* cloned into MCS2 pACYCDuet EcoRV | NNNNNNGATATCATGCTATACAAGGGGTGTCT |
| 3’ *tacA* cloned into MCS2 pACYCDuet XhoI | NNNNNNCTCGAGTTACACGTCCCACTGAGGTT |
| 5’ Upstream *tacA* | NNGCTCTTCNTTCAATTAACAAGGTCTGTACAGGGATGCTATCAG |
| **Sequence verification primers** | |
| 5’ T7 Foward | TAATACGACTCACTATAGGG |
| 3’ T7 Reverse | GCTAGTTATTGCTCAGCGG |
| 5’ pBAD sequencing primer | CTGTTTCTCCATACCCGTT |
| 3’ pBAD sequencing primer | GGCTGAAAATCTTCTCT |
| 5’ *tacT* sequencing primer | TCAACTTAACCTTAGAGCTAAGGA |
| 3’ *tacT* sequencing primer | GCTCACTTTGTACTGACCCC |
| 5’ *tacA* sequencing primer | CACGGCTTTCATATAGTGGAGCG |
| 3’ *tacA* sequencing primer | GCAAGACGGGCAAGTATAATGACAGGG |
| **EMSA primers** | |
| 5’ Probe 1, Probe 2 EMSA primer | CCTTGTTGAACTCAGTAAACCG |
| 3’ Probe 1, Probe 3, Probe 5 EMSA primer | ACCCCTTGTATAGCATTTGC |
| 3’ Probe 2 EMSA primer | \| TGAATACTGATGTTCGCTT \| \| --- \| |
| 5’ Probe 3, Probe 4 EMSA primer | ACGACGACTTGATGTATAC |
| 3’ Probe 4 EMSA primer | ACCATGTATAGCAAACGG |
| 5’ Probe 5 EMSA primer | TGGTTGTGCTATTCTTGTA |
| **RT-qPCR primers** | |
| 5’ *tacA* RTqPCR | CCTTGACCGCCGTGTATTT |
| 3’ *tacA* RTqPCR | GGTTTCCTTGCCAGCAGTT |
| 5’ *rpoB* RTqPCR | AGTCGACCTGAGCACTTCA |
| 3’ *rpoB* RTqPCR | CAAACACTGGTGTGGCAATC |
| 5’ *gyrB* RTqPCR | CCGTTGGATCACGAGTTTG |
| 3’ *gyrB* RTqPCR | AACGCGTCCTCTTCAATCAG |
